# Pathogenesis of Lethal Aspiration Pneumonia in *Mecp2*-null Mouse Model for Rett Syndrome

**DOI:** 10.1038/s41598-017-12293-8

**Published:** 2017-09-20

**Authors:** Hiroshi Kida, Tomoyuki Takahashi, Yuki Nakamura, Takashi Kinoshita, Munetsugu Hara, Masaki Okamoto, Satoko Okayama, Keiichiro Nakamura, Ken-ichiro Kosai, Takayuki Taniwaki, Yushiro Yamashita, Toyojiro Matsuishi

**Affiliations:** 1 Division of Gene Therapy and Regenerative Medicine, Cognitive and Molecular Research Institute of Brain Diseases, Kurume University, 67 Asahi-machi, Kurume, 830-0011 Fukuoka Japan; 2 Division of Respirology, Neurology, and Rheumatology, Department of Medicine, Kurume University school of Medicine, Kurume, Japan; 3 Department of Pediatrics and Child Health, Kurume University school of Medicine, Kurume, Japan; 4 Department of Anatomy, Kurume University school of Medicine, Kurume, Japan; 5 Department of Gene Therapy and Regenerative Medicine, Advanced Therapeutics Course, Kagoshima University Graduate School of Medical and Dental Sciences, Kagoshima, Japan; 6 Research Center for Children, Research Center for Rett syndrome, St. Mary’s Hospital, Kurume, Japan

## Abstract

Rett syndrome (RTT) is a neurodevelopmental disorder mainly caused by mutations in the gene encoding the transcriptional regulator Methyl-CpG-binding protein 2 (MeCP2), located on the X chromosome. Many RTT patients have breathing abnormalities, such as apnea and breathing irregularity, and respiratory infection is the most common cause of death in these individuals. Previous studies showed that MeCP2 is highly expressed in the lung, but its role in pulmonary function remains unknown. In this study, we found that MeCP2 deficiency affects pulmonary gene expression and structures. We also found that *Mecp2*-null mice, which also have breathing problems, often exhibit inflammatory lung injury. These injuries occurred in specific sites in the lung lobes. In addition, polarizable foreign materials were identified in the injured lungs of *Mecp2*-null mice. These results indicated that aspiration might be a cause of inflammatory lung injury in *Mecp2*-null mice. On the other hand, MeCP2 deficiency affected the expression of several neuromodulator genes in the lower brainstem. Among them, neuropeptide substance P (SP) immunostaining was reduced in *Mecp2*-null brainstem. These findings suggest that alteration of SP expression in brainstem may be involved in autonomic dysregulation, and may be one of the causes of aspiration in *Mecp2*-null mice.

## Introduction

Rett syndrome (RTT, MIM #312750) is a neurodevelopmental disorder characterized by intellectual disability, loss of expressive speech, deceleration of head growth, and loss of acquired skills^1–6^. Other clinical symptoms include gait abnormalities, seizures, sleep problems, teeth grinding, chewing difficulties, air swallowing, irregular breathing, and cardiac abnormalities. Mutations of *Mecp2*, located on the X chromosome, are found in about 90% of individuals meeting the diagnostic criteria for classical RTT^7,8^. Consistent with this, knockout mouse models with a disrupted *Mecp2* gene mimic many key clinical features of RTT and provide an excellent platform for understanding the pathogenesis of RTT^2,9,10^.

Among the most prominent clinical features of RTT are breathing irregularities, including breath holding, apnea, apneusis, hyperventilation, and rapid shallow breathing^11–15^. The respiratory dysfunctions observed in RTT patients have been replicated in several mutant mouse models^16–18^. In addition, several studies suggested that breathing abnormalities in RTT can be attributed to severe autonomic and brainstem dysfunction. However, recent studies suggest that the respiratory dysfunction observed in RTT may also result from abnormalities of the pulmonary system itself^19–24^. High-resolution computed tomography (HRCT) imaging studies showed that pulmonary lesions including centrilobular nodules, bronchial wall thickening, and patchy ground-glass opacities are present in about 50% of typical patients with RTT^20^. In addition, a diffuse inflammatory infiltrate of terminal bronchioles and alveoli was observed in 50% of the *Mecp2*-mutant mice examined^23^. On the other hand, MeCP2 is strongly expressed in the adult mouse lung, and regulated throughout alveolarization in the rat lung^22^. In addition, MeCP2 may be essential for myofibroblast differentiation and pulmonary fibrosis^21,24^. Although these studies provided important information, the significance of pulmonary expression of MeCP2 remains unknown. In particular, the pathogenesis of inflammatory lung injury in MeCP2-deficient mice and RTT patients remains to be elucidated.

In this study, we investigated developmental mechanisms of inflammatory lung injury and examined the effects of MeCP2 deficiency in the respiratory system itself. Our results demonstrate that MeCP2 deficiency affects pulmonary gene expression and lung structures. In addition, aspiration pneumonia might be a cause of inflammatory lung injury in the *Mecp2*-null mouse model of RTT.

## Results

### Breathing problems and histologic features of lungs in *Mecp2*-deficient mice

In our laboratory, *Mecp2*-null mice exhibited reduced spontaneous movement between 3 and 8 weeks of age, and died at approximately 7–10 weeks^25^. At the late stages of disease (7–10 weeks), most *Mecp2*-null mice exhibited reduced mobility and severe weakness. During this stage, *Mecp2*-null mice exhibited breathing problems (22/29 cases, 75.9%), and death could occur at any time. Some deaths were sudden, but most of *Mecp2*-null mice became symptomatic for a brief period of time (1–3 days) prior to death.

**Figure 1:**
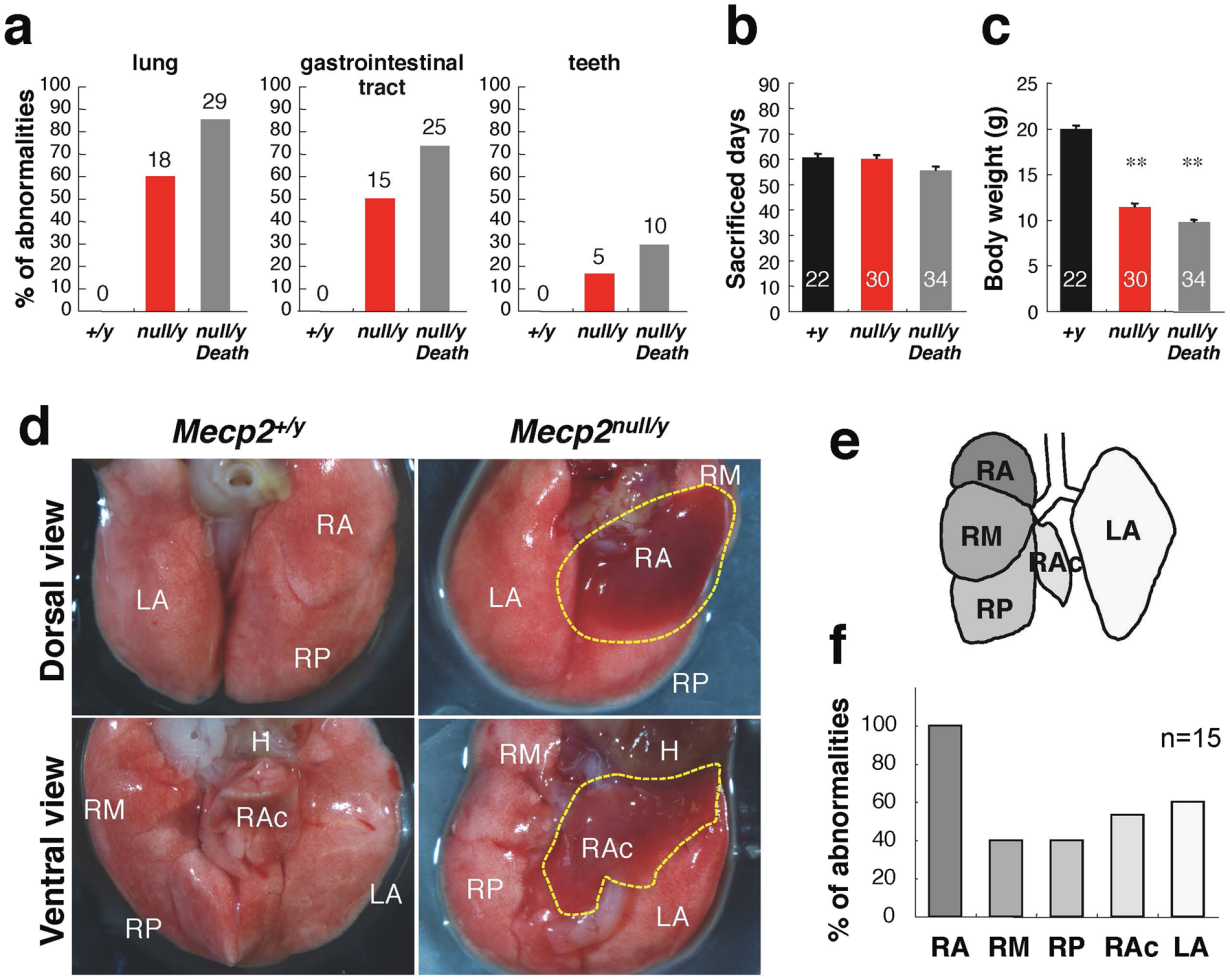
Lung abnormalities in *Mecp2*-null mice. (**a**) Incidence of abnormalities in the indicated organs and tissues in wild-type (*Mecp2* ^+/*y*^, black columns; n = 22), *Mecp2*-null (*Mecp2* ^*null*/*y*^, red columns; n = 30), and dead *Mecp2*-null (*Mecp2* ^*null*/*y*^ *Death*, gray columns; n = 34) mice. (**b**) Age (days) at which *Mecp2*-null mice died or were sacrificed. Data are expressed as means ± SE. (**c**) Body weight (**g**), measured at sacrifice. Data are expressed as means ± SE. **p < 0.01 versus wild-type mice. (**d**) Gross morphology of lung from wild-type (left panels) and *Mecp2*-null (right panels) mice. Dashed yellow lines indicate injured area. RA, right anterior; RM, right middle; RP, right posterior; RAc, right accessory; and LA, left anterior lobe. (**e**) Schematic diagram indicating mouse lung lobes (ventral view). (**f**) Incidence of abnormalities in each pulmonary lobe of *Mecp2*-null lungs (n = 15).

*Mecp2*-null mice were sacrificed just before death from disease, and their lungs were dissected and examined (Fig. 1). At sacrifice (60.5 ± 1.4 days old for wild type, n = 22; 60.1 ± 1.5 days old for *Mecp2*-null, n = 30), body weight was significantly lower in *Mecp2*-null mice (19.9 ± 0.4 g in wild type, n = 22; 11.3 ± 0.4 g in *Mecp2*-null, n = 30; p < 0.01) (Fig. 1b,c). Macroscopic observation revealed that 60% (18/30) of *Mecp2*-null mice had lung abnormalities (Fig. 1a). These lung abnormalities were seen more often in the anterior right pulmonary lobe (15/15 cases, 100%) than in the middle or posterior right lobe (6/15 cases, 40%) (Fig. 1d–f). Postmortem examination also revealed that over 80% (29/34 cases) of dead mutant mice had lung abnormalities (Fig. 1a and Supplementary Fig. S1). These results indicate that lung abnormalities in *Mecp2*-null mice occurred in specific pulmonary lobes.

Extra-pulmonary findings included gas (air bubbles) filling the digestive organs of *Mecp2*-null mice, whereas those of wild-type mice were empty or filled with food (Fig. 1a and Supplementary Fig. S2). The accumulation of air bubbles in the digestive tract of *Mecp2*-null mice was observed in mice with severe cases of breathing problems. It is unknown whether this observation is a primary or secondary effect of the physical state of the animal just prior to analysis. However, aerophagia is common in RTT patients, and leads to significant gaseous distension of the intestinal tract^26^. These observations indicate that the swelling of the digestive tract in *Mecp2*-null mice was likely to have been caused by the misguided intake of aspirated air into the digestive tract. In addition, *Mecp2*-null mice occasionally showed malocclusion (overgrowth of incisor teeth and misplaced midline) and fractured teeth. These tooth phenotypes were observed in 29% of the deceased *Mecp2*-null mice (Fig. 1a and Supplementary Fig. S1d). However, necropsy failed to reveal any obvious alteration in *Mecp2*-null larynx, other than size and thickness, possibly due to the lower body size/weight of mutant mice (Supplementary Fig. S3).

**Figure 2:**
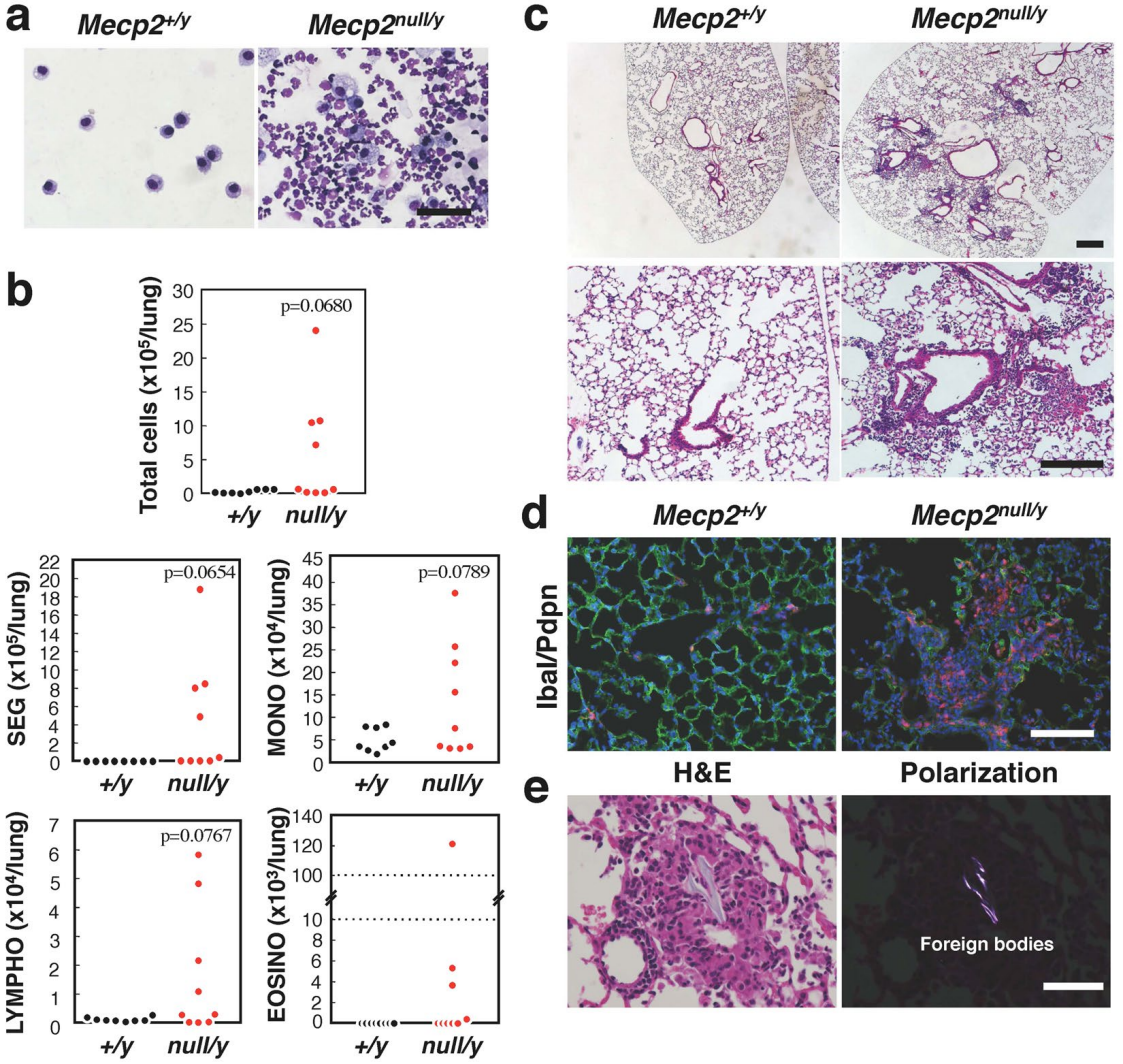
Lung inflammation in *Mecp2*-null mice. (**a**) Morphology of BAL cells from wild-type (left panel) and *Mecp2*-null (right panel) mice. Cells in BALFs were centrifuged onto glass slides, dried in air, and stained with Wright-Giemsa. Scale bar indicates 200 μm. (**b**) Cell populations in BALFs. Plots showed the indicated cell counts in BALFs obtained from wild-type (n = 8) and *Mecp2*-null mice (n = 9). SEG, Segmented Neutrophils; MONO, Monocytes; LYMPHO, Lymphocytes; and EOSINO, Eosinophils. (**c**) Representative H&E stained sections of the right anterior lobe in wild-type (left panel) and *Mecp2*-null (right panel) lungs. Scale bars, 200 μm. (**d**) Representative immunofluorescence images showing the distribution of IbaI signals in wild-type (left panels) and *Mecp2*-null (right panels) lung tissues. Cryosections of right anterior lung lobes obtained from 8-week-old wild-type and *Mecp2*-null mice were immunostained for IbaI (red) and Pdpn (green), and counterstained with Hoechst 33342 (blue). Scale bar, 100 μm. (**e**) H&E-stained sections of *Mecp2*-null lungs observed under bright-field (left panel) and polarization (right panel). Scale bar, 50 μm.

Next, we investigated whether the population of inflammatory cells was increased in the lungs of *Mecp2*-null mice (Fig. 2). For this purpose, bronchoalveolar lavage fluid (BALF) was isolated from wild-type (n = 8) and *Mecp2*-null (n = 9) mice at 7–10 weeks of age (Fig. 2a,b). In four cases (44.4%), the number of total cells, lymphocytes, monocytes, eosinophils, and segmented neutrophils were obviously elevated in *Mecp2*-null mice relative to wild-type controls. In addition, in five (55.6%) *Mecp2*-null mice, segmented neutrophils represented over 50% of the cells in the BALF fluid (Supplementary Fig. S4). These results indicate that nearly half of *Mecp2*-null mice had inflammation in the lung.

We then performed histological analysis of lung abnormalities in *Mecp2*-null mice (Fig. 2c–e). Histological analysis of injured anterior right pulmonary lobes in *Mecp2*-null mice demonstrated that large numbers of infiltrating cells were present in the epithelial layer of the alveoli, as well as the alveolar space, indicating severe inflammation in the lungs (Fig. 2c). In addition, Iba1-positive macrophages were clearly observed in the injured lung of *Mecp2*-null mice (Fig. 2d and Supplementary Fig. S5). By contrast, Iba1-positive macrophages were rarely observed in the lungs of wild-type mice. Thus, histological analysis also indicate that inflammation was induced in the injured lungs of *Mecp2*-null mice. Furthermore, we observed polarizable foreign material in injured lungs of *Mecp2*-null mice (Fig. 2e). This material was irregularly sized and shaped, ranging from translucent to pale blue. Foreign materials in abnormal lungs suggest that the inflammatory lung injury in *Mecp2*-null mice is induced by aspiration.

In addition to the gross phenotype, mice from each group were examined by routine blood biochemistry. Blood plasma measurements revealed modest changes in a small number of markers in *Mecp2*-null mice, but we observed no life-threatening abnormal laboratory values in either healthy or abnormal *Mecp2*-null mice. (Supplementary Table S1).

Serum Amyloid P Component (SAP) and C-reactive protein (CRP), which belong to the short pentaxin subfamily, are major acute-phase plasma proteins in mouse and human, respectively^27^. In lung-injured *Mecp2*-null mice, the plasma level of SAP was significantly higher (>30-fold) than that in wild-type and *Mecp2*-null mice (Supplementary Table S1). The elevation of SAP may indicate the presence of a severe systemic inflammatory response in *Mecp2*-null mice. These data may also help to explain the severe systemic inflammation that causes death in lung-injured *Mecp2*-null mice.

### Effects of MeCP2 deficiency on lung structures

**Figure 3:**
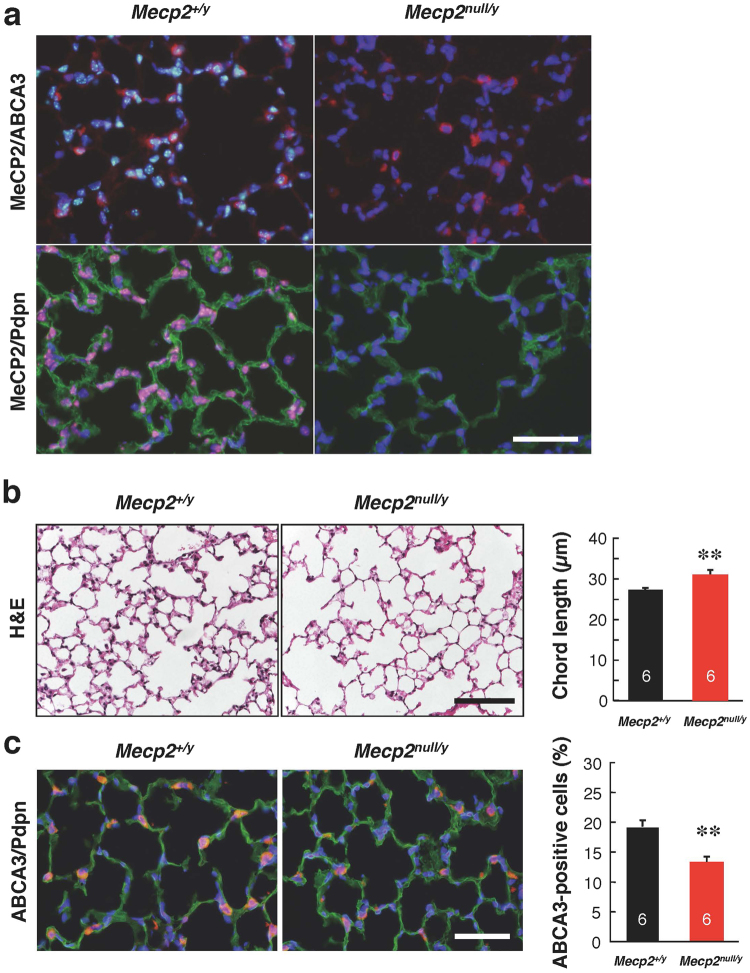
Histological analysis of lungs from *Mecp2*-null mice. (**a**) Representative immunofluorescence images showing the distribution of MeCP2, ABCA3, and Pdpn signals in wild-type and *Mecp2*-null lungs. Cryosections of right anterior lobes obtained from 8-week-old wild-type (left panel) and *Mecp2*-null (right panel) mice were immunostained for MeCP2 (green, upper panel or red, lower panel), ABCA3 (red), and Pdpn (green), and then counterstained with Hoechst 33342 (blue). MeCP2 signals were detected in wild-type lung tissues. Scale bar, 50 μm. (**b**) Representative H&E stained lung sections of wild-type (left panel) and *Mecp2*-null (right panel) mice. Scale bar, 100 μm. The graph at right shows the mean alveolar chord lengths in wild-type (n = 6) and *Mecp2*-null (n = 6) lungs. (**c**) Representative immunofluorescence images showing the distribution of ABCA3 and Pdpn signals in wild-type (left panels) and *Mecp2*-null (right panels) lungs. Scale bar, 50 μm. The graph shows the percentage of ABCA3-positive cells in wild-type (n = 6) and *Mecp2*-null (n = 6) mice lungs. Data are expressed as means ± SE. *p < 0.05 and **p < 0.01 versus wild-type mice.

Immunofluorescence staining revealed MeCP2 signals in wild-type lung tissues (Fig. 3). The MeCP2-positive cells were colocalized with Podoplanin (Pdpn) or ATP-Binding Cassette A3 (ABCA3), which are the alveolar type I (ATI) or type II (ATII) cell markers, respectively (Fig. 3a and Supplementary Figs S6 and S7).

Following hematoxylin and eosin (H&E) staining, alveolar structures were generally preserved in *Mecp2*-null mice without inflammatory lung injury. However, the number of alveoli that were contiguous with alveolar ducts was reduced in some areas. We morphometrically quantified the alveolar airspace in tissue sections of *Mecp2*-null and wild-type lungs (Fig. 3b). The mean values of the alveolar airspace were significantly greater in *Mecp2*-null mice than in wild-type mice. These findings indicate that *Mecp2*-null mice exhibited emphysema-like changes in the lung.

ATII cells synthesize the pulmonary surfactant, which makes important contributions to maintaining alveolar and airway stability^28^. ABCA3 is a lamellar body–associated lipid transport protein required for normal synthesis and storage of pulmonary surfactant in ATII cells^28,29^. Immunostaining of ABCA3 revealed that *Mecp2*-null lungs contained fewer ATII cells than wild-type lung (Fig. 3c and Supplementary Fig. S8). These results suggest that MeCP2 deficiency alters the number of ABCA3-positive ATII cells and induces emphysema-like structural changes in the lung.

### Alterations of endogenous gene expression in MeCP2-deficient lung

**Figure 4:**
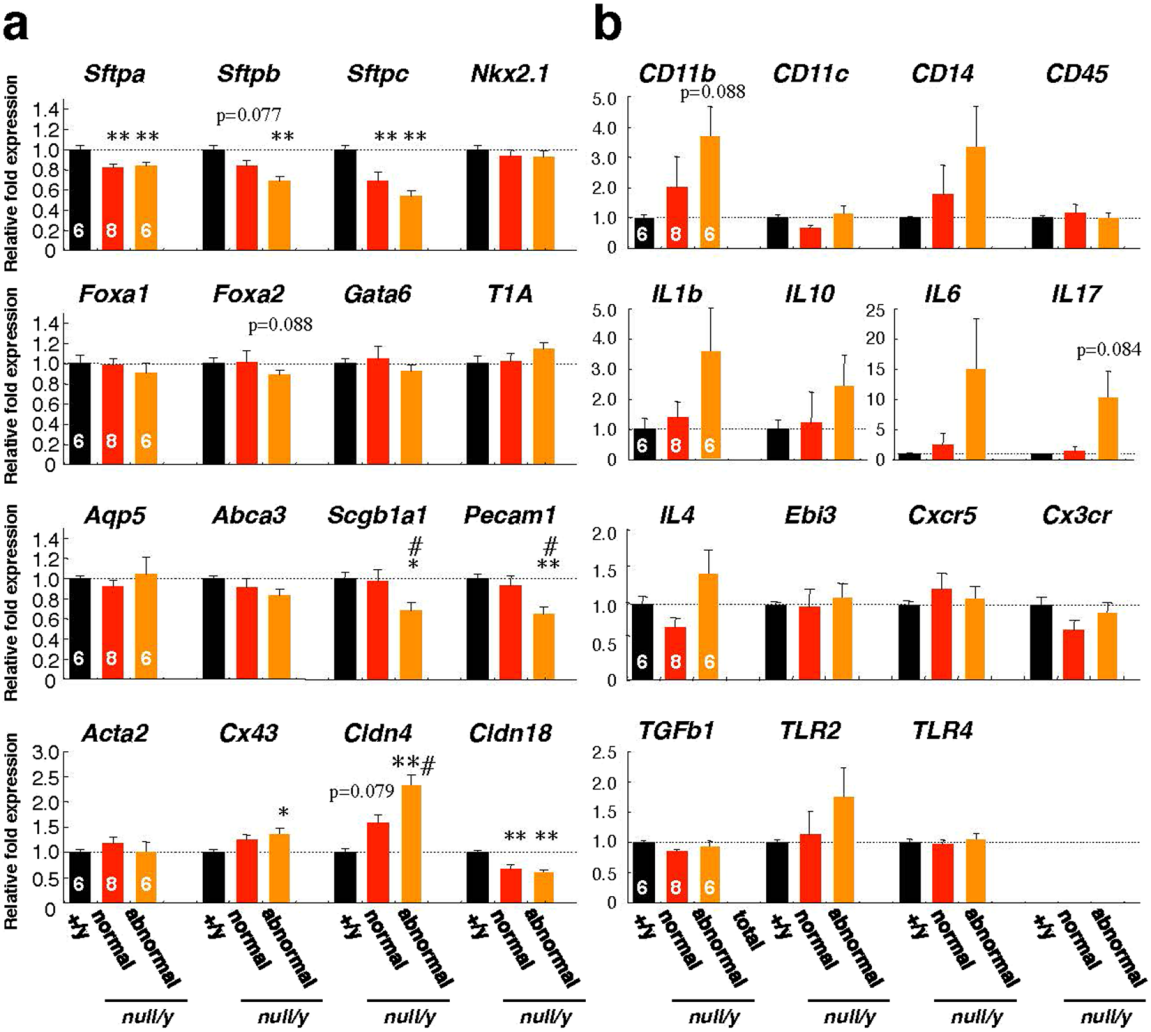
Endogenous gene expression in wild-type and *Mecp2*-null mice lungs. Whole lungs of wild-type and *Mecp2*-null mice were harvested, and qRT-PCR was performed using primers against the indicated genes (**a**, lung-specific marker genes; **b**, inflammation-related genes). The graphs show the relative levels of the indicated mRNAs in wild-type (black columns, n = 6), normal *Mecp2*-null (red columns, n = 8), and abnormal *Mecp2*-null (orange columns, n = 6) lungs harvested from 7–10 week-old mice. The level of each mRNA was normalized to the level of *Rps18*. All data were normalized to the corresponding values from wild-type mice, defined as 1.0. Data are expressed as means ± SE. *p < 0.05 and **p < 0.01 versus wild-type samples; ^#^p < 0.05 versus *Mecp2*-null samples.

To determine the effects of MeCP2 deficiency on lung gene expression *in vivo*, we performed quantitative real-time reverse transcription–polymerase chain reaction (qRT-PCR) analyses of lung genes, using total RNA prepared from whole lung taken from 8–10-week-old mice (Fig. 4). The mRNA levels of *Sftpa*, *Sftpb*, *Sftpc*, and *Cdln18* were significantly lower in *Mecp2*-null mice than in wild-type mice (Fig. 4a). In addition, the mRNA levels of *Scgb1a1* and *Pecam1* were significantly lower in abnormal *Mecp2*-null lungs. By contrast, *Cldn4* mRNA levels were higher in *Mecp2*-null mice, and the mRNA levels of *Cldn4* and *Cx43* were significantly higher in the *Mecp2*-null abnormal lungs than in wild-type lungs. These results indicate that loss of MeCP2 leads, either directly or indirectly, to transcriptional dysregulation of these genes in the adult mouse lung.

Next, we investigated the expression of genes involved in the inflammatory response in injured *Mecp2*-null lung (Fig. 4b). The mRNA levels of *CD11b* and *CD14* in *Mecp2*-null lungs were elevated, but not statistically significant, compared to wild-type lungs. In abnormal *Mecp2*-null lungs, *CD11b*, *CD14*, *IL1b*, *IL6*, *IL17*, *IL4*, and *TLR2* mRNA levels were higher, but not statistically significant, compared to wild-type lungs. These results suggest that the expression of inflammatory response genes is associated with the pathology of aspiration pneumonia in *Mecp2*-null mice.

### Intranasal administration of adenoviral vector results in transduction of the lung in awake *Mecp2*-null mice

In animal models, intranasal administration is currently the most widely used method for delivery of drugs, vaccines, or pathogen challenges targeting the lungs. Several studies indicate that the use of anesthesia overrides the swallowing reflex, and this approach has been used in mice to promote more efficient delivery of materials into the lungs^30–32^. By contrast, in awake mice, delivery of materials into the lungs may be prevented by protective systems such as swallowing or the cough reflex.

To investigate potential dysfunctions in the system protecting the respiratory tract in *Mecp2*-null mice, we intranasally administered adenoviral vectors (Ad.LacZ) expressing the lacZ reporter gene to wild-type and *Mecp2*-null mice (Supplementary Fig. S9). In addition, to discriminate the results from endogenous beta-galactosidase activity, we administered mock adenoviral vectors (Ad.dE1.3) lacking a gene intranasally.

In the initial studies, adenoviral vectors (Ad-LacZ or Ad.dE1.3) were administered intranasally to wild-type and *Mecp2*-null mice under anesthesia. Mice given Ad.dE1.3 expressed no LacZ in the lungs (Supplementary Fig. S9a). By contrast, LacZ reporter genes were strongly expressed in the lungs of the wild-type and *Mecp2*-null mice given Ad.LacZ. Because aspiration of an intranasal solution into the lungs is caused by disturbed upper airway reflexes during anesthesia, LacZ expression was also observed in the lungs.

Next, Ad.LacZ was administered intranasally to wild-type (n = 14) and *Mecp2*-null (n = 14) mice without anesthesia (Supplementary Fig. S9b). In comparison with the anesthesia group, faint expression of LacZ was observed in one (7.1%) wild-type mouse given Ad.LacZ without anesthesia. By contrast, expression of LacZ gene was detected in nine (64.3%) *Mecp2*-null mice. In addition, expression of LacZ in the lungs was mainly detected in the right anterior lobe and/or the upper fields of the left lobe in *Mecp2*-null mice. These results suggest that aspiration in *Mecp2*-null mice may be related to a dysfunction in protective systems such as swallowing and the coughing reflex.

### Alterations of endogenous gene expression in *Mecp2*-deficient brainstems

**Figure 5:**
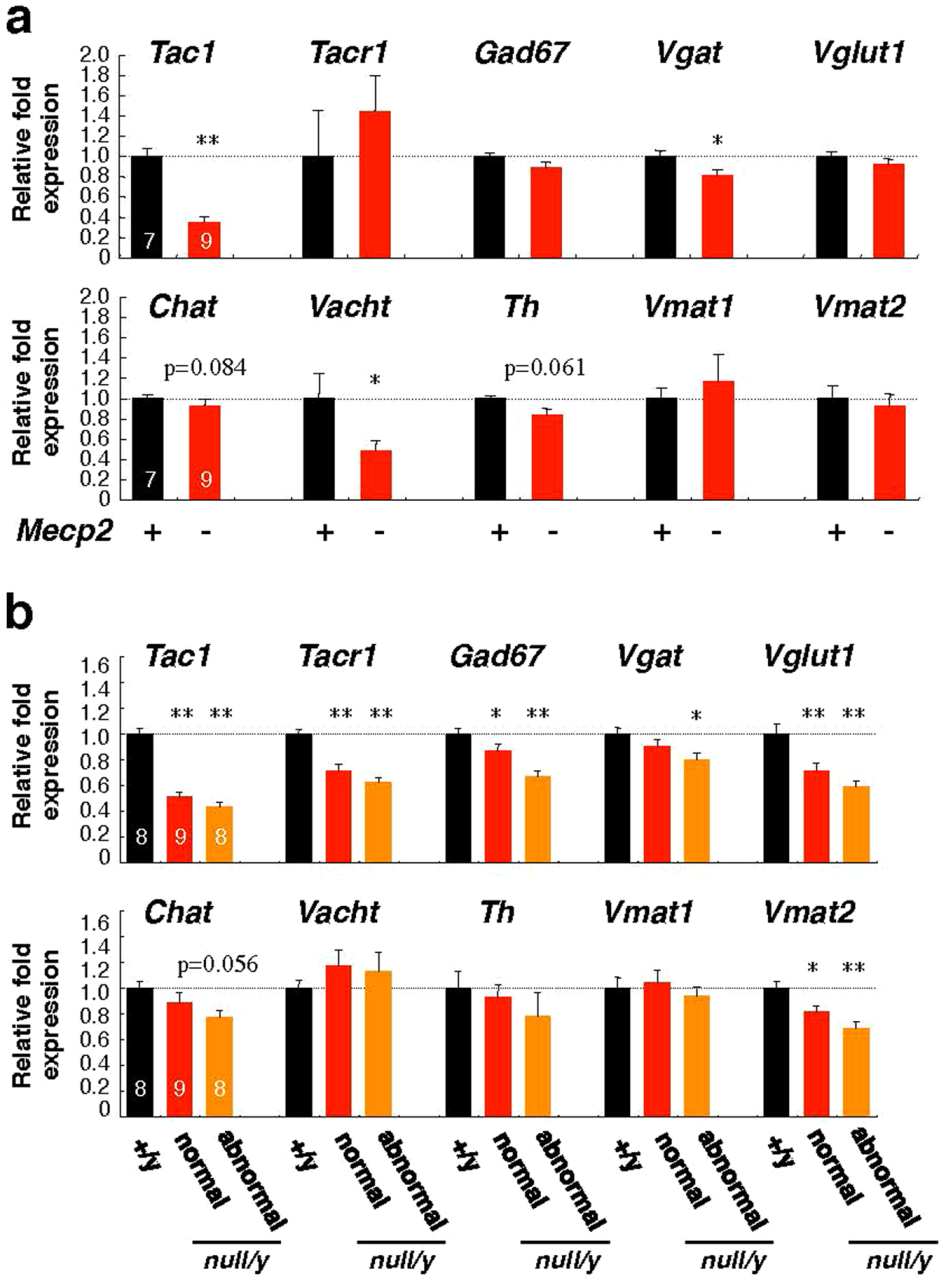
Endogenous gene expression in wild-type and *Mecp2*-null mice brainstem. Whole brain and brainstem of wild-type and *Mecp2*-null mice were harvested, and qRT-PCR was performed using primers for the indicated genes (**a**, whole brain; **b**; brainstem). The graphs show the relative tissue levels of the indicated mRNAs in wild-type (black columns, n = 8), normal *Mecp2*-null (red columns, n = 9), and abnormal *Mecp2*-null (orange columns, n = 8) organs harvested from 7–10 week-old mice. The level of each mRNA was normalized to the corresponding level of *Rps18*. All data were normalized to the value from wild-type mice, defined as 1.0. Data are expressed as means ± SE. *p < 0.05 and **p < 0.01 versus wild-type mice samples.

The brainstem is the key brain region involved in the regulation of breathing, sneezing, and swallowing^33,34^. Therefore, we investigated the expression of genes involved in neurotransmission in the lower brainstem, including the pons and the medulla oblongata (Fig. 5). In the whole brain, the mRNA levels of *Tac1*, *Vgat*, and *Vacht* were significantly lower in *Mecp2*-null mice than in wild-type mice (Fig. 5a). The mRNA levels of *Th* were lower in *Mecp2*-null mice, although the differences were not statistically significant.

In lower brainstem samples, the mRNA levels of *Tac1*, *Tacr1*, *Gad67*, *Vglut1*, and *Vmat2* were significantly lower in *Mecp2*-null mice than in wild-type mice (Fig. 5b). In addition, the mRNA levels of *Vgat* were significantly lower in abnormal *Mecp2*-null mice. Furthermore, a decrease in *Tac1*, *Tacr1*, *Gad67*, *Vglut1*, and *Vmat2* mRNA levels was associated with lung abnormalities in *Mecp2*-null mice, although the differences were not statistically significant. These results indicate that MeCP2 deficiency impaired expression of genes involved in the production of key neurotransmitters, including substance P, γ-aminobutyrate (GABA), glutamate, and monoamines, in the lower brainstem. These data indicate that dysfunction of the neurotransmitter system in the brainstem may lead to a malfunction in swallowing and the airway defense systems, in association with aspiration, in *Mecp2*-null mice.

### Analysis of the neuropeptide substance P expression in *Mecp2*-deficient brainstems

**Figure 6:**
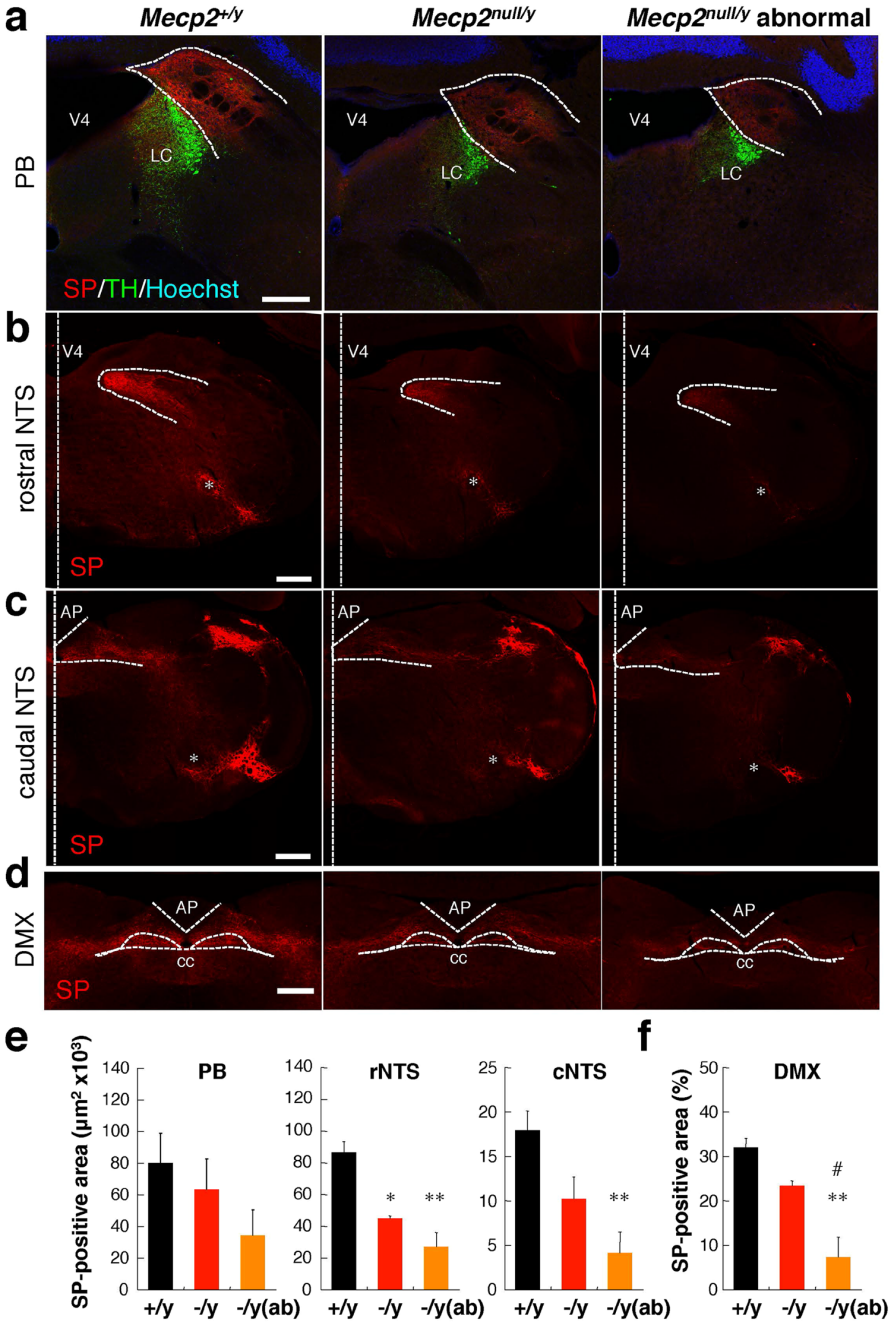
SP immunoreactivity in wild-type and *Mecp2*-null mouse brainstem. Representative images of distribution of SP-ir structures in the brainstem: PB in the pons (**a**), and rostral NTS (**b**), caudal NTS (**c**), and DMX (**d**) in the medulla oblongata. Cryosections of brainstem obtained from 7–10-week-old wild-type (left panels), normal *Mecp2*-null (middle panels), and abnormal *Mecp2*-null (right panels) mice were immunostained for SP (red) and TH (green), and counterstained with Hoechst 33342 (blue, merged image panels) (Supplementary Figure S10). Dorsal is up. Vertical dotted lines indicate the median. White dotted outlines identify areas of the PB (**a**), NTS (**b** and **c**), and DMX (**d**). V4: fourth ventricle; PB: parabrachial nucleus; LC: locus coeruleus; NTS: nucleus of the solitary tract; AP: area postrema; DMX: dorsal motor nucleus of vagus nerve; cc: central canal. Asterisks indicate the nucleus ambiguous (AMB). Scale bars, 300 μm. (**e**) Comparison of the areas of SP-ir in the indicated regions of the brainstem in wild-type (black columns, n = 4), normal *Mecp2*-null (red columns, n = 3), and abnormal *Mecp2*-null (orange columns, n = 4) mice (Supplementary Figures S11 and S12). (**f**) Comparison of the percentage of the SP-ir area in DMX regions in wild-type (black columns, n = 4), normal *Mecp2*-null (red columns, n = 3), and abnormal *Mecp2*-null (orange columns, n = 4) mice. Data are expressed as means ± SE. *p < 0.05 and **p < 0.01 versus wild-type samples; ^#^p < 0.05 versus *Mecp2*-null samples.

In our qRT-PCR analysis, the mRNA level of *Tac1*, which encodes the neuropeptide substance P (SP), was markedly reduced in *Mecp2*-null mice. To define potential sites in which dysregulation of neuropeptide SP expression may contribute to brainstem dysfunction, we compared the brainstem distribution of neuropeptide SP between wild-type and *Mecp2*-null mice (Fig. 6 and Supplementary Figs 10–12).

The distribution of SP-immunoreactivities (SP-ir) identified in this study are consistent with the previously reported distribution^35–38^. SP-ir was mostly found in puncta, fibers, and/or varicosities rather than in cell bodies; these structural features resembled those reported in the rat brainstem^35^.

In wild-type mice, SP-ir were clearly detected in parabrachial area (PB) of the pons, processes throughout the nucleus of the solitary tract (NTS), the spinal nucleus of the trigeminal nerve (SPV), and the spinal tract of the trigeminal nerve (sptV). SP-ir structures in PB were distributed in close proximity to the locus coeruleus (LC), stained with an antibody against tyrosine hydroxylase (TH) (Fig. 6a). In the rostral NTS region, SP-ir structures were dense in the medial region, but the density gradually decreased in the lateral regions (Fig. 6b). In the caudal medulla, SP-ir structures were detected in the lateral portions of the caudal NTS (Fig. 6c). In the dorsal motor nucleus of the vagus nerve (DMX), the density of SP-ir structures were similar to the densities seen in the medial subdivision of the NTS (Fig. 6d). In addition, a high density of SP-ir structures was found in the SPV and sptV. On the other hand, faint and diffuse staining was present in other regions, including the nucleus raphe pallidus (RPA) and nucleus raphe obscurus (RO).

Previous studies showed that SP-immunoreactive neuronal cell bodies are also present in regions of the brainstem such as the NTS, RPA, RO, and area postrema (AP)^35,37^. Blockade of axonal transport by colchicine pretreatment is necessary for localization of significant SP-ir in neuronal cell bodies^35,37^. Under our experimental conditions, we could detect only a very small amount of immunostaining in neuronal bodies, possibly because we did not use colchicine.

In *Mecp2*-null mice, although the regional pattern of SP-ir was very similar, the density of SP-ir was lower than in wild-type mice in all areas. In addition, semiquantitative measurements revealed that the area of SP-ir structures was significantly reduced in the rostral NTS, caudal NTS, and DMX of abnormal *Mecp2*-null mice (Fig. 6e and f). These results indicate that MeCP2 deficiency affects the expression/distribution of SP in the lower brainstem of autonomic control centers. These findings also suggest that alteration of SP expression/distribution in the brainstem may lead to dysfunction of autonomic systems, leading to aspiration, in *Mecp2*-null mice.

## Discussion

Many RTT patients have breathing problems, including hyperventilation and breath holding^12,13,15^. The influence of these breathing problems on overall health and survival is not well understood, and further research in this area is required. On the other hand, the Australian Rett Syndrome Database (ARSD) indicates that pneumonia is the most common cause of death^39,40^. Therefore, the influence of respiratory infection in RTT patients may also need to be closely monitored. In fact, lower respiratory tract infections including pneumonia occur in nearly 20% of RTT patients. Most RTT patients have shallow breathing, which is one reason why some RTT patients catch colds very easily, often developing into pneumonia. Furthermore, the strongest risk factor for pneumonia is aspiration. This is an important issue among RTT patients because it might be caused by autonomic dysfunctions related to swallowing, coughing, and gastroesophageal reflux, as well as scoliosis^39–42^.

The major complication associated with aspiration is pulmonary infection^43–45^. As a clinical feature, aspiration can lead to the development of site-specific lobar or segmental pneumonia^43^. In fact, the usual sites for aspiration pneumonia are the apical and posterior segments of the lower lobe of the right lung. In this study, we observed site-specific pulmonary injury in *Mecp2*-null mice. In addition, severe inflammation and polarizable foreign materials were identified in injured lungs of *Mecp2*-null mice. Additionally, abnormalities of the teeth in some *Mecp2*-null mice may have lead to oral dysphagia due to the risk of overly large or coarse boluses. These results indicated that the respiratory inflammation and damage in *Mecp2*-null mice were induced by aspiration.

Swallowing and coughing are complex processes involving a sequence of intricate, timed manoeuvres coordinated by brainstem neural networks and multiple neuromuscular systems (including mouth, pharynx, oesophagus, and diaphragm)^33,34^. In RTT, impairment of the autonomic nervous system is reflected in respiratory and cardiac dysfunctions^1,13,15,46^. The brainstem is the key part of the brain involved in autonomic regulation, and the immaturity of the brainstem has been proposed as a reason for autonomic dysfunctions observed in RTT^16,18,47–51^. Therefore, it is not surprising that these choreographies are disturbed by poor coordination of neuromuscular conditions in RTT patients.

In fact, neurochemical studies in RTT patients indicated alterations in various neurotransmitter systems, including acetylcholine, dopamine, serotonin, glutamate, substance P, and various tropic factors^6,52–57^. Several studies reported that disordered GABA and biogenic amine signals in brain respiratory networks cause irregular breathing and apnea in RTT patients and mouse models^12,58–60^. In this study, we also demonstrated that the mRNA levels of *Tac1*, *Tacr1*, *Vgat*, *Vglut1*, and *Vmat2* in the brainstem were significantly reduced in *Mecp2*-null mice. In addition, we found that the decrease in the levels of those transcripts tended to depend on the presence of lung abnormalities. These findings suggest that disturbances in various neurotransmitter systems of the lower brainstem may lead to aspiration in *Mecp2*-null mice.

Among these genes, the mRNA level of *Tac1*, which encodes the neuropeptide substance P (SP), was markedly reduced in *Mecp2*-null mice. Consistent with this, we previously reported that the SP level in cerebrospinal fluid (CSF) is significantly reduced in RTT patients^54^. In addition, SP immunoreactivity is significantly lower in multiple brain regions of RTT patients^55^. In this study, immunostaining for SP in abnormal *Mecp2*-null brainstem also revealed a significant reduction in SP staining in several regions, including the NTS and DMX. Evidence from animal models suggests that the SP is involved in cardiorespiratory regulation, gastrointestinal regulation, and the cough reflex at the brainstem level, including the NTS^36,61–63^. For example, SP evokes cardiovascular responses that may be dependent upon changes in baroreflex function in the NTS^63^. In rat, SP applied to the NTS induces a significant increase in minute ventilation^62^, and microinjection of SP into the DMX decreases intragastric pressure, antral motility, and gastric acid secretion^36^. Furthermore, direct microinjections of SP into the NTS decreases the electrical threshold for cough evoked from the tracheal mucosa by electrical stimulation or by citric acid^62^. In addition, local application of SP to rabbit NTS potentiates cough motor responses and increases cough number, peak abdominal activity, and expiratory rise rate in response to stimulus^62^. On the other hand, previous studies in sudden perinatal implicated the trigeminal ganglion along with its nerve and SP/NK-1R expression alteration as a possible pathophysiological mechanism^64^. Collectively, these findings suggest that alteration of SP expression/distribution in the brainstem makes a crucial contribution to autonomic dysregulation in *Mecp2*-null mice.

However, SP neurons are also present in many forebrain regions that make descending input to the NTS^35,36^. In addition, SP is widely distributed throughout the peripheral nervous systems and organs of vertebrates^36,61^. Hence, further studies of the role of SP in forebrain regions and peripheral organs may provide insight into the complex pathogenesis of RTT.

On the other hand, during swallowing, the respiratory tract is protected not only by physiological reflexes, but also by anatomical structures^34,65,66^. We found that the macroscopic structures of the larynx were generally maintained in *Mecp2*-null mice. In mice, the velum and epiglottis are tightly apposed during rest breathing through the nose, protecting the larynx from the normal path of bolus flow during swallowing^66^. However, mice are preferential, but not obligate, nasal breathers, and they can switch to an oral mode of breathing with no apparent discomfort, as reported in previous studies investigating the effects of nasal occlusion^67^. These studies indicate that the larynx may not be always protected from penetration/aspiration due to unexpected events, illness, or disease. In addition, the risk of aspiration is believed to be related not only to dysphagia during eating but also to several gastrointestinal symptoms such as gastroesophageal reflux (GER)^68,69^. In fact, GER or esophageal dysmotility can result in aspiration, even in healthy subject confused by the sudden appearance of gastroesophageal contents in the pharynx^69^. Aerophagia can also be responsible for symptoms mimicking GER^70^. Many Rett patients experience gastrointestinal abnormalities, such as GER^26,41,42^. Therefore, we cannot rule out the possibility that aspiration in *Mecp2*-null mice could be related to multiple gastrointestinal symptoms, including GER and aerophagia. These finding suggest that, in *Mecp2*-null mice and RTT patients, gastrointestinal symptoms are among the risk factors of aspiration, as is the case with dysfunction of the swallowing and cough reflexes.

In the lung itself, a previous study implicated MeCP2 in myofibroblast differentiation and bleomycin-induced pulmonary fibrosis^21,24^. In addition, intrauterine growth restriction (IUGR), which increases the risk of lung disease in the immediate neonatal period as well as later in life, alters MeCP2 expression in the lung^22^. Those findings indicated that MeCP2 could mediate various physiological processes, including the development, homeostasis, and repair of the lung.

Our qRT-PCR analysis showed that transcript levels of several lung-specific genes (such as *Sftpa*, *Sftpb*, *Sftpc*, *Cldn4*, and *Cldn8* genes) were altered in *Mecp2*-null mice lungs. These results indicated that MeCP2 directly or indirectly regulates the expressions of various genes that play roles in the lung, including ATII cells.

Histopathological analyses revealed that *Mecp2*-null mice exhibited emphysema-like changes in the lung. In addition, immunostaining revealed a significant reduction of ABCA3-positive ATII cells in *Mecp2*-null lungs. The reduction in the number of ABCA3-positive ATII cells, which are vital for alveolar development, homeostasis, and injury repair, may underlie the emphysema-like changes in *Mecp2*-null lungs^28,29^. These results suggest that MeCP2 deficiency causes structural or pathological changes that may have facilitated the development of emphysema in *Mecp2*-null lungs. This is of particular interest for future studies because emphysema-like changes might explain the role of MeCP2 in the lung itself.

On the other hand, multiple factors, including oxidative stress and inflammation, cannot be excluded as contributors to altered gene expression and structural abnormalities. Previous studies have indicated that oxidative stress and chronic inflammation contribute to the pathologic alterations of the lung in emphysema^71^. In addition, respiratory dysfunction in RTT patients and model mice leads to significant hypoxia and oxidative stress, both of which are functionally detrimental to many tissues and organs^72,73^. Therefore, the altered gene expression and structural abnormalities can be attributed, at least in part, to several pathological conditions in *Mecp2*-null mice.

In conclusion, we demonstrated that *Mecp2*-null mice have inflammatory lung injuries, which occurred in specific sites in the lung lobes. In addition, polarizable foreign materials were identified in injured lungs of *Mecp2*-null mice. These data strongly indicate that *Mecp2*-null mice have aspiration pneumonia. We also found that MeCP2 deficiency affects the expression of several neuromodulator genes in the lower brainstem, suggesting that lower brainstem dysfunction plays a predominant role in the lethal aspiration pneumonia observed in *Mecp2*-null mice. Among these neuromodulators, the neuropeptide SP immunostaining in *Mecp2*-null brainstem was significantly reduced in several regions, including the NTS and DMX. These results indicated that alterations of SP expression/distribution in the brainstem may be involved in autonomic dysregulation, and thus contribute to aspiration in *Mecp2*-null mice. Our studies will help predict which RTT patients are at risk for aspiration pneumonia, and may be useful for developing new therapeutics, as well as for understanding the importance of suitable medical interventions.

## Methods

### Animal

*Mecp2* ^−/+^ female mice (B6.129P2(C)-*Mecp2* ^tm1.1Bird^/J strain) were purchased from the Jackson Laboratory (Bar Harbor, ME, USA) and mated with wild-type C57BL/6 male mice^9^. Genotyping was performed by PCR analysis of genomic DNA according to the protocol provided by the supplier (http://jaxmice.jax.org/pub-cgi/protocols/protocols.sh?objtype=protocol&protocol_id=598). DNA samples were extracted from the tails of newborn mice after digestion with proteinase K. All experiments were performed in accordance with the National Institutes of Health Guidelines for the Care and Use of Laboratory Animals and were approved by the Animal Research Committee of Kurume University.

### Isolation of Bronchoalveolar Lavage Fluid (BALF)

Mice were sacrificed under anesthesia by intraperitoneal injection of sodium pentobarbital. A tubing adaptor was inserted into the trachea, and the lungs were washed three times with 2 ml PBS. The recovered bronchoalveolar lavage fluid (BALF) was evaluated with a hemocytometer. Aliquots of cells were centrifuged onto glass slides, dried in air, and stained with Wright-Giemsa. Cell populations were then calculated as reported previously^74^.

### Blood analysis

Blood samples were collected via cardiac punctures of the left ventricle under anesthesia. Heparin plasma samples were separated by centrifugation at 1000 × g for 10 minutes, and the supernatant was transferred to a new tube. Aliquots of plasma samples were frozen at −80 °C. Biochemical analysis of plasma was performed by the Nagahama Life-science Laboratory of Oriental Yeast Co., Ltd. (Nagahama, Shiga, Japan). Endogenous murine SAP was measured using the mouse pentraxin2/SAP Quantikines ELISA kit (R&D Systems Inc., Minneapolis, MN, USA).

### Histology

For histological analysis, mice were sacrificed under deep anesthesia by intraperitoneal injection of sodium pentobarbital. After the thorax was opened, the lungs were immediately fixed by intratracheal instillation of 10% buffered formalin for 15–20 minutes at a constant pressure of 25 cm H_2_O. After gross examination, the extracted tissues were placed in 10% buffered formalin and further fixed for at least 24 hours. The tissues were then embedded in paraffin, cut into 4-μm thick sections, and stained with hematoxylin and eosin (H&E)^74^. Mean alveolar chord length was estimated by using a computerized color image analysis software system (WinROOF Version 5.0, Mitani Co., Fukui, Japan), as previously described^75^.

To reveal foreign bodies in the lungs, H&E stained sections were imaged with an Olympus BX51 fluorescence microscope fitted with an analyzer (UANT) and polarizer (U-POT).

### Immunohistochemistry

Under deep anesthesia, lungs were immediately fixed by intratracheal instillation of 4% paraformaldehyde for 15–20 minutes at a constant pressure of 25 cm H_2_O^25,75^. The extracted tissues were placed into 4% paraformaldehyde and fixed for an additional 4 hours. For cryosections (8 μm), mouse lungs were equilibrated in a 30% sucrose solution and mounted in FSC 22® frozen section compound. (Leica, Wetzlar, Germany). Sections were fixed with 4% paraformaldehyde at RT for 10 min, subjected to immunofluorescence staining with primary antibodies against MeCP2 (1:500 dilution; D4F3, Cell Signaling, Beverly, MA, USA), ABCA3 (1:500; 3C9, Covance, Berkeley, CA, USA), Podoplanin (1:500; 8.1.1, BioLegend), AQP5 (1:500; Alomone Labs, Jerusalem, Israel), and Iba1 (1:500; Wako Pure Chemical Industries, Osaka, Japan), and visualized with secondary fluorescent antibodies. Sections were counterstained with Hoechst 33342 and examined on a Zeiss Axioskop2 Plus microscopy system (Carl Zeiss, Oberkochen, Germany). Photomicrographs were captured using an AxioCam HRc digital camera or a Biorevo BZ-9000 fluorescence microscope (KEYENCE Co., Osaka, Japan); images were analyzed using the BZ-II application.

For brain samples, coronal sections were cut though the brainstem at a thickness of 25 μm using a cryostat, and then mounted. Brain sections were incubated with 0.05% Triton X-100 at RT for 15 min, subjected to immunofluorescence staining with primary antibodies against substance P (SP) (1:200 dilution; MAB356, Millipore, Billerica, MA, USA) and tyrosine hydroxylase (TH) (1:250; AB152, Millipore, Billerica, MA, USA), and then visualized with fluorescent secondary antibodies. Brain anatomic regions including the NTS were defined based on morphology with reference to images in Paxinos and Franklin^76,77^. Co-staining with antibodies against NeuN (1:500; MABN140, Millipore, Billerica, MA, USA), somatostatin (1:200; MAB354, Millipore, Billerica, MA, USA), choline acetyltransferase (ChAT) (1:200; AB144P, Millipore, Billerica, MA, USA), or TH was also used help define anatomic regions within the sections. Sections were counterstained with Hoechst 33342 and examined on a Zeiss Axioskop2 Plus microscopy system (Carl Zeiss, Oberkochen, Germany). Photomicrographs were captured on a Biorevo BZ-X710 fluorescence microscope (KEYENCE Co., Osaka, Japan), and the images were processed and reconstructed in BZ-X Analyzer software. All semiquantitative measurements were carried out in comparable areas under the same optical and light conditions. A total of six images (left and right regions in three serial brain sections) per region in each animal were analyzed to measure the areas of SP-ir structures.

### PCR analysis

Extraction of total RNA and quantitative real-time PCR (qRT-PCR) analysis were carried out as described previously^25^. The qRT-PCR was performed on a LightCycler Nano (Roche Diagnostics, Basel, Switzerland) using FastStart Essential DNA Green Master (Roche Diagnostics). Expression levels of all genes were normalized against the level of Rps18 mRNA. Primer sequences are listed in the Supplementary Table 2.

### Intranasal administration of adenoviral vectors

Replication-defective recombinant adenoviral vectors (Ads), Ad.LacZ and Ad.dE1.3, which respectively express LacZ or no gene under the transcriptional control of the CA promoter, were prepared as described previously^78,79^.

Seven- to ten-week-old wild-type and *Mecp2*-null (at the late stage of disease) mice were intranasally administered 15 μl (2 × 10^8^ pfu) of Ads (Ad.LacZ or Ad.dE1.3), with or without anesthesia^30–32^. For anesthesia-induced aspiration, mice were anesthetized with chloral hydrate (200–400 mg/kg) until loss of the foot reflex, placed in a supine position, and given Ads by nasal instillation. For intranasal Ads delivery while the animals were awake, mice were hand-restrained, placed in a supine position, and given virus through the nostrils. All mice were subsequently sacrificed 24 hours later, and lungs were immediately fixed by intratracheal instillation of 4% paraformaldehyde for 15–20 minutes at a constant pressure of 25 cm H_2_O. The expression of LacZ was analyzed by X-gal staining, as previously described^79^.

### Statistical analysis

Quantitative results are expressed as means ± standard error (SE). Statistical significance was determined using Student’s t-test. One-way ANOVA analysis and Tukey–Kramer test were used for multiple comparisons. p < 0.05 was considered to be statistically significant. All statistical analysis was performed with the JMP Pro 11 software (SAS Institute, Cary, NC, USA).

## Electronic supplementary material

Supplementary Information
