# Supplementary material for: Pathogenesis of Lethal Aspiration Pneumonia in Mecp2-null Mouse Model for Rett Syndrome
Source: Sci Rep. 2017 Sep 20;7:12032. doi: 10.1038/s41598-017-12293-8 (PMC5607245; doi:10.1038/s41598-017-12293-8)
Supplement: Supplementary file 1 — Supplementary Information [file 41598_2017_12293_MOESM1_ESM.pdf]

Supplementary Information

Pathogenesis of Lethal Aspiration Pneumonia in *Mecp2*-null Mouse Model for Rett Syndrome

Hiroshi Kida, Tomoyuki Takahashi, Yuki Nakamura, Takashi Kinoshita, Munetsugu Hara, Masaki Okamoto, Satoko Okayama, Keiichiro Nakamura, Ken-ichiro Kosai, Takayuki Taniwaki, Yushiro Yamashita, Toyojiro Matsuishi

Inventory of supplemental Items

-Supplementary Tables

Related to Figure 1 and 2
- Table S1

Related to Methods
- Table S2

-Supplementary Figures and Figure Legends

Related to Figure 1:
- Supplementary Figure S1
- Supplementary Figure S2
- Supplementary Figure S3

Related to Figure 2:
- Supplementary Figure S4
- Supplementary Figure S5

Related to Figure 3:
- Supplementary Figure S6
- Supplementary Figure S7
- Supplementary Figure S8

Supplementary Figure S9

Related to Figure 6:
- Supplementary Figure S10
- Supplementary Figure S11
- Supplementary Figure S12

Table S1. Results of blood biochemistry analysis of *Mecp2*-null mice.

Heart blood was sampled at 7–10 weeks for biochemical analysis. TP, total protein; ALB, albumin; BUN, blood urea nitrogen; CRE, creatinine; IP, inorganic phosphorus; AST, aspartate transaminase; ALT, alanine transaminase; LDH, lactate dehydrogenase; AMY, amylase; T-CHO, total cholesterol; TG, triglyceride; HDL-C, HDL cholesterol; T-BIL, total bilirubin; SAP, serum amyloid P component. Data are expressed as means ± SE. Groups were compared using one-way ANOVA and the Tukey–Kramer test. *, $p < 0.05$ and **, $p < 0.01$ versus wild-type (*Mecp2*$^{+/y}$) mice. $^{\#}$, $p < 0.05$ and $^{\#\#}$, $p < 0.01$ versus *Mecp2*-null (*Mecp2*$^{null/y}$) mice.

	***Mecp2*$^{+/y}$**	***Mecp2*$^{null/y}$**	***Mecp2*$^{null/y}$ (abnormal)**
	**n = 16**	**n = 8**	**n = 8**
**TP (g/dL)**	4.93±0.06	5.05±0.07	4.95±0.11
**ALB (g/dL)**	3.58±0.04	3.88±0.06*	3.48±0.15$^{\#}$
**BUN (mg/dL)**	25.56±1.10	36.55±2.61**	35.75±2.52**
**CRE (mg/dL)**	0.11±0.003	0.10±0.003	0.11±0.004
**IP (mg/dL)**	11.31±0.24	9.03±0.48**	7.13±0.43$^{**\ \#\#}$
**AST (IU/L)**	47.75±1.56	63±5.89	104.25±7.83$^{**\ \#\#}$
**ALT (IU/L)**	23.88±0.78	29.75±5.35	73.75±13.69$^{**\ \#\#}$
**LDH (IU/L)**	130.50±5.48	143.75±12.14	168.00±12.09*
**AMY (IU/L)**	1931.88±60.27	2200.75±97.13	5446.25±2602.54
**T-CHO (mg/dL)**	75.38±1.60	70.75±2.95	100.00±7.76$^{**\ \#\#}$
**TG (mg/dL)**	45.75±5.46	52±9.42	30.75±6.91
**HDL-C (mg/dL)**	41.88±0.92	36.75±1.19	39.5±2.72
**T-BIL (mg/dL)**	0.0625±0.005	0.045±0.007	0.083±0.042
**SAP (μg/mL)**	0.95±0.07	1.84±0.47	32.29±11.41$^{**\ \#\#}$

Table S2. Primers used for quantitative real-time PCR.

**Quantitative real time PCR**			
Gene	Forward Primer	Reverse Primer	Ta (°C)
Sftpa	5'-CCAGGGTTTCCAGCTTACCT-3'	5'-GATCCTTGCAAGCTGAGGAC-3'	60
Sftpb	5'-CCTGGAACACCAGTGAACAG-3'	5'-CATGTGCTGTTCCACAAACTG-3'	60
Sftpc	5'-CTCGTTGTCGTGGTGATTGT-3'	5'-TTTCTGAGTTTCCGGTGCTC-3'	60
Nkx2.1	5'-ACAGCCAAGCAAATTCAACC-3'	5'-TAAGCTTGGGAACCCATTTG-3'	58
Foxa1	5'-ACAGGGTTGGATGGTTGTGT-3'	5'-GTCCGCGTAGTAGCTGTTCC-3'	62
Foxa2	5'-TTTGGGAGAGCTTTGAGGAA-3'	5'-TGTGGCCCATCTATTTAGGG-3'	58
Gata6	5'-CTACACAAGCGACCACCTCA-3'	5'-GTAGAGGCCGTCTTGACCTG-3'	60
T1A	5'-AGCCGCTGTAGAACCAAGAA-3'	5'-CTGAGAAGCAGAAGGCAGGT-3'	60
Aqp5	5'-GGAAGAAGACCATCGAGCTG-3'	5'-GAGGTGCTCCAAACTCTTCG-3'	60
Abca3	5'-ACCCTGAAGAAACGGAAGGT-3'	5'-CTCCGACTGGATCTTCAAGC-3'	58
Scgb1a1	5'-GTCATGCTGTCCATCTGCTG-3'	5'-GGGATGCCACATAACCAGAC-3'	60
Pecam	5'-GTCATGGCCATGGTCGAGTA-3'	5'-TCCTCGGCGATCTTGCTGAA-3'	57
Acta2	5'-GAGAAGCCCAGCCAGTCG-3'	5'-CTCTTGCTCTGGGCTTCA-3'	58
Cx43	5'-GAGAGCCCGAACTCTCCTTT-3'	5'-TGGGCACCTCTCTTTCACTT-3'	61
Cldn4	5'-ACCTCGTAGCAACGACAAGC-3'	5'-CAGAGTGGCCCACCTTACAC-3'	60
Cldn18	5'-GGCCATACTTCACCATCCTG-3'	5'-AGATGGACACGAGGATACCG-3'	60
CD11b	5'-GCATCAACAACATTGACTTTCA-3'	5'-CTCGTCCGAGTACTGCATCA-3'	60
CD11c	5'-ATTTTCACATGGACGGTGCT-3'	5'-CAGTTGCCTGTGTGATAGCC-3'	60
CD14	5'-CCACCGCTGTAAAGGAAAGA-3'	5'-CAGAAGCAACAGCAACAAGC-3'	60
CD45	5'-CTTACCTGCTCGCACCACT-3'	5'-AGCAGCGTGGATAACACACC-3'	59
IL1b	5'- GACCTTCCAGGATGAGGACA-3'	5'-TAATGGGAACGTCACACACC-3'	60
IL10	5'-ACCAGCTGGACAACATACTG-3'	5'-CGCATCCTGAGGGTCTTCAG-3'	60
IL6	5'-GTCACAGAAGGAGTGGCTA-3'	5'-AGAGAACAACATAAGTCAGATACC-3'	60
IL17	5'-CTCCAGAAGGCCCTCAGACTAC-3'	5'-GGGTCTTCATTGCGGTGG-3'	60
IL4	5'-ACTCTTTCGGGCTTTTCGAT-3'	5'-TTGCATGATGCTCTTTAGGC-3'	58
Ebi3	5'-CTTCCTGTCACTTGCCCTCT-3'	5'-ATACCGAGAAGCATGGCATT-3'	60
Cxcr5	5'-GACTCCTTACCACAGTGCACCTT-3'	5'-TGGAAACGGGAGGTGAACCA-3'	60
Cx3cr1	5'-AAGTTCCCTTCCCATCTGCT-3'	5'-AATGTCGCCCAAATAACAGG -3'	60
TGFb1	5'-CAACGCCATCTATGAGAAAACC-3'	5'-AAGCCCTGTATTCCGTCTCC-3'	60
TLR4	5'-ATCTGAGCTTCAACCCCTTG-3'	5'-AGAGGTGGTGTAAGCCATGC-3'	58
Tac1	5'-TCGATGCCAACGATGATCTA-3'	5'-AAAGAACTGCTGAGGCTTGG-3'	60
Tacr1	5'-GCTGCTCTCTTCGCCAGTAT-3'	5'-CCAGGACCCAGATGACAAAG-3'	60
Gad67	5'-CTTCTTCCGGATGGTCATCTC-3'	5'-ACGAGCAACATGCTATGGTC-3'	60
Vgat	5'-ACTGCGACGATCTCGACTTT-3'	5'- ATCTTGGGTTTGTCGTGACC -3'	60
Vglut1	5'-CCAGCATCTCTGAGGAGGAG-3'	5'-GGCTGAGAGATGAGGAGCAG-3'	60
Chat	5'-GCCAATCCATTCCCACTGAC-3'	5'-CATCCAAGACAAAGAACTGG-3'	60
Vacht	5'-CTAATGCCAGCGCCTACTTG-3'	5'-AGGCAAATAGCACGCCTATC-3'	60
Th	5'-TGCTGTTCTCAACCTGCTCTT-3'	5'-AGTGGTGGATTTTGGCTTCA-3'	60
Vmat1	5'-TGGTGGTGTTTGTGGCTCTA-3'	5'-GGACCCCTAAGCAGAGAAGA-3'	60
Vmat2	5'-AGACCATGTGTTCCCGAAAG-3'	5'-AGAAGAGCACACAGCCACCT-3'	60
Rps18	5'-TTCTGGCCAACGGTCTAGACAAC-3'	5'-CCAGTGGTCTTGGTGTGCTGA-3'	58

Supplementary Figure Legends

Supplementary Figure 1.

Lung and teeth abnormalities in dead *Mecp2*-null mice.

Postmortem examinations in *Mecp2*-null mice were performed immediately after death from disease. (**a**) Dorsal (left) and ventral (right) views of the whole lung from dead *Mecp2*-null mice. Dashed yellow lines indicate injury area. RA, right anterior; RM, right middle; RP, right posterior; RAc, right accessory; and LA, left anterior lobe. (**b**) Representative H&E stained sections of the right anterior lobes in the *Mecp2*-null mice lungs. Scale bars indicate 200 μm. (**c**) H&E-stained sections of *Mecp2*-null lungs observed under bright-field (left panel) and polarization (right panel). Scale bars indicate 50 μm. (**d**) Representative gross morphology of teeth. In wild-type mice, symmetric mandibular incisors were longer than maxillary incisors (left panel). Incisors of *Mecp2*-null were misaligned and overgrown (middle panel) and/or broken (right panel). Tooth abnormalities were observed in ~30% of *Mecp2*-null mice.

Supplementary Figure 2.

Macroscopic observation of the gastrointestinal tract in *Mecp2*-null mice.

Macroscopic views of the gastrointestinal tract after laparotomy in wild-type (**a**) and *Mecp2*-null (**b**) mice. Representative images (right panels) of the entire gastrointestinal tract in wild-type and *Mecp2*-null mice at the age of 8 weeks. The boxed areas in (**b**) are shown below at a higher magnification. Air bubbles were observed in the digestive organs of *Mecp2*-null mice, whereas stomachs in wild-type mice were filled with food. Air bubbles are indicated by white arrowheads. lu; lung, li; liver, st; stomach.

Supplementary Figure 3.

Macroscopic observation of the larynx in wild-type and *Mecp2*-null mice.

Representative images of the larynx in wild-type (left panels) and abnormal *Mecp2*-null (right panels) mice at the age of 9–10 weeks. (**a**) The velum (white asterisk) and

epiglottis are tightly apposed in mice, preventing visualization of the epiglottis and vocal folds via a transoral approach. **(b)** The epiglottis (black asterisk) was gradually visualized after mechanical separation of the velum and glottis using surgical instruments. **(c)** The glottis (white arrowhead) was visualized in stages by mechanical separation of the velum and glottis using surgical instruments. The macroscopic structures of the larynx were generally maintained in *Mecp2*-null mice.

Supplementary Figure 4.

Bronchoalveolar lavage fluid (BALF) analysis of wild-type and *Mecp2*-null mice.

Cell populations in BALFs. Plots showed the percentage of indicated cells in BALFs obtained from wild-type (n = 8) and *Mecp2*-null mice (n = 9). Data are expressed as means ± SE. *, $p < 0.05$ versus wild-type mice. SEG, Segmented Neutrophils; MONO, Monocytes; LYMPHO, Lymphocytes; and EOSINO, Eosinophil.

Supplementary Figure 5.

Immunofluorescence of Iba I in wild-type and *Mecp2*-null lungs.

Representative immunofluorescence images showing the distribution of Iba I signals in wild-type and *Mecp2*-null lung tissues. Cryosections of right anterior lung lobe obtained from 8-week-old wild-type (left panels) and *Mecp2*-null (right panels) mice were immunostained for Iba I (red) and Pdpn (green), and counterstained with Hoechst 33342 (blue). Scale bars indicate 100 μm.

Supplementary Figure 6.

Double-immunofluorescence staining for MeCP2 and ABCA3 in wild-type and *Mecp2*-null lungs.

Representative immunofluorescence images showing the distribution of MeCP2 and ABCA3 signals in wild-type and *Mecp2*-null lungs. Cryosections of right anterior lobes obtained from 8-week-old wild-type (left panels) and *Mecp2*-null (right panels) mice

were immunostained for MeCP2 (green) and ABCA3 (red), and counterstained with Hoechst 33342 (blue). MeCP2 signals were detected in wild-type lung tissues. Double-label immunofluorescence also confirmed that nuclear MeCP2 signals were detected in ABCA3-positive ATII cells. Scale bars indicate 50 μm.

Supplementary Figure 7.

Double-immunofluorescence staining for MeCP2 and Pdpn in wild-type and *Mecp2*-null lungs.

Representative immunofluorescence images showing the distribution of MeCP2 and Pdpn signals in wild-type and *Mecp2*-null lungs. Cryosections of right anterior lobes obtained from 8-week-old wild-type (left panels) and *Mecp2*-null (right panels) mice were immunostained for MeCP2 (red) and Pdpn (green), and counterstained with Hoechst 33342 (blue). Double-label immunofluorescence indicted that nuclear MeCP2 signals were detected in Pdpn-positive ATI cells. Scale bars indicate 50 μm.

Supplementary Figure 8.

Double-immunofluorescence staining for ABCA3 and Pdpn in wild-type and *Mecp2*-null lungs.

Representative immunofluorescence images showing the distribution of ABCA3 and Pdpn signals in wild-type and *Mecp2*-null lungs. Cryosections of right anterior lobes obtained from 8-week-old wild-type (left panel) and *Mecp2*-null (right panel) mice were immunostained for ABCA3 (red), Pdpn (green), and counterstained with Hoechst 33342 (blue). Scale bars indicate 50 μm.

Supplementary Figure 9.

LacZ gene expression in lungs after intranasal administration.

Macroscopic views of lungs of wild-type and *Mecp2*-null mice after intranasal administration of adenoviral vectors (Ad.LacZ or Ad.dE1.3), with or without anesthesia. (**a**) X-gal–stained whole lungs from wild-type mice after intranasal administration of

Ad.dE1.3 (upper panels) and Ad.LacZ (lower panels) with anesthesia. X-gal (blue) staining across several lobes occurred in an airway-associated and a patchy-diffuse pattern. Blue staining of the trachea was attributed to endogenous β-galactosidase activity, as similar staining was detected in Ad.dE1.3-treated mice. **(b)** Macroscopic views of lungs after intranasal administration of Ad.LacZ to wild-type (upper panels) and *Mecp2*-null mice (lower panels) without anesthesia. In the wild-type group, no or only a faint LacZ signal was observed in lung lobes. In the *Mecp2*-null group, blue staining of lung lobes reflected expression of LacZ (arrowhead).

Supplementary Figure 10.

SP immunoreactivity in wild-type and *Mecp2*-null mouse brainstem.

(a) Illustration of the medulla oblongata: rostral NTS, left panels; caudal NTS, right panels. Medial is to the left; dorsal is up. **(b)** Representative images of distribution of SP-ir structures in the medulla oblongata. Cryosections of brainstem obtained from 7–10-week-old wild-type (top panels), normal *Mecp2*-null (middle panels), and abnormal *Mecp2*-null (bottom panels) mice were immunostained for SP (red) and TH (green), and counterstained with Hoechst 33342 (blue, merged image panels). NTS: nucleus of the solitary tract; RO: nucleus raphe obscurus; RPA: nucleus raphe pallidus; py: pyramid; AMB: nucleus ambiguous; AP: area postrema; DMX: dorsal motor nucleus of vagus nerve; SPV: spinal nucleus of the trigeminal; sptV: spinal tract of trigeminal nerve. Scale bars, 300 μm.

Supplementary Figure 11.

SP immunoreactivity in the rostral NTS of wild-type and *Mecp2*-null mouse brainstem.

Representative images of the distribution of SP-ir structures in the rostral NTS. **(a)** Cryosections of brainstem obtained from 7–10-week-old wild-type (left panels), normal *Mecp2*-null (middle panels), and abnormal *Mecp2*-null (right panels) mice were immunostained for SP (red) **(b)** and TH (green), and counterstained with Hoechst 33342

(blue, merged image panels). **(c)** Images from **(b)** were processed and reconstructed in the BZ-X Analyzer software, and the areas of SP-ir structures were determined by measuring positively stained areas based on preset thresholds. Areas selected as SP-immunostained are pseudo-colored yellow. Scale bars, 150 μm.

Supplementary Figure 12.

SP immunoreactivity in the caudal NTS of wild-type and *Mecp2*-null mouse brainstem.

Representative images of the distribution of SP-ir structures in the caudal NTS. **(a)** Cryosections of brainstem obtained from 7–10-week-old wild-type (left panels), normal *Mecp2*-null (middle panels), and abnormal *Mecp2*-null (right panels) mice were immunostained for SP (red) **(b)** and TH (green), and counterstained with Hoechst 33342 (blue, merged image panels). White dotted outlines indicate areas of the AP and DMX. AP: area postrema; DMX: dorsal motor nucleus of vagus nerve; cc: central canal. **(c and d)** Images from **(b)** were processed and reconstructed in the BZ-X Analyzer software, and the areas of SP-ir structures were measured. Areas selected as SP-immunostained are pseudo-colored yellow. Scale bars, 100 μm.

Figure S1

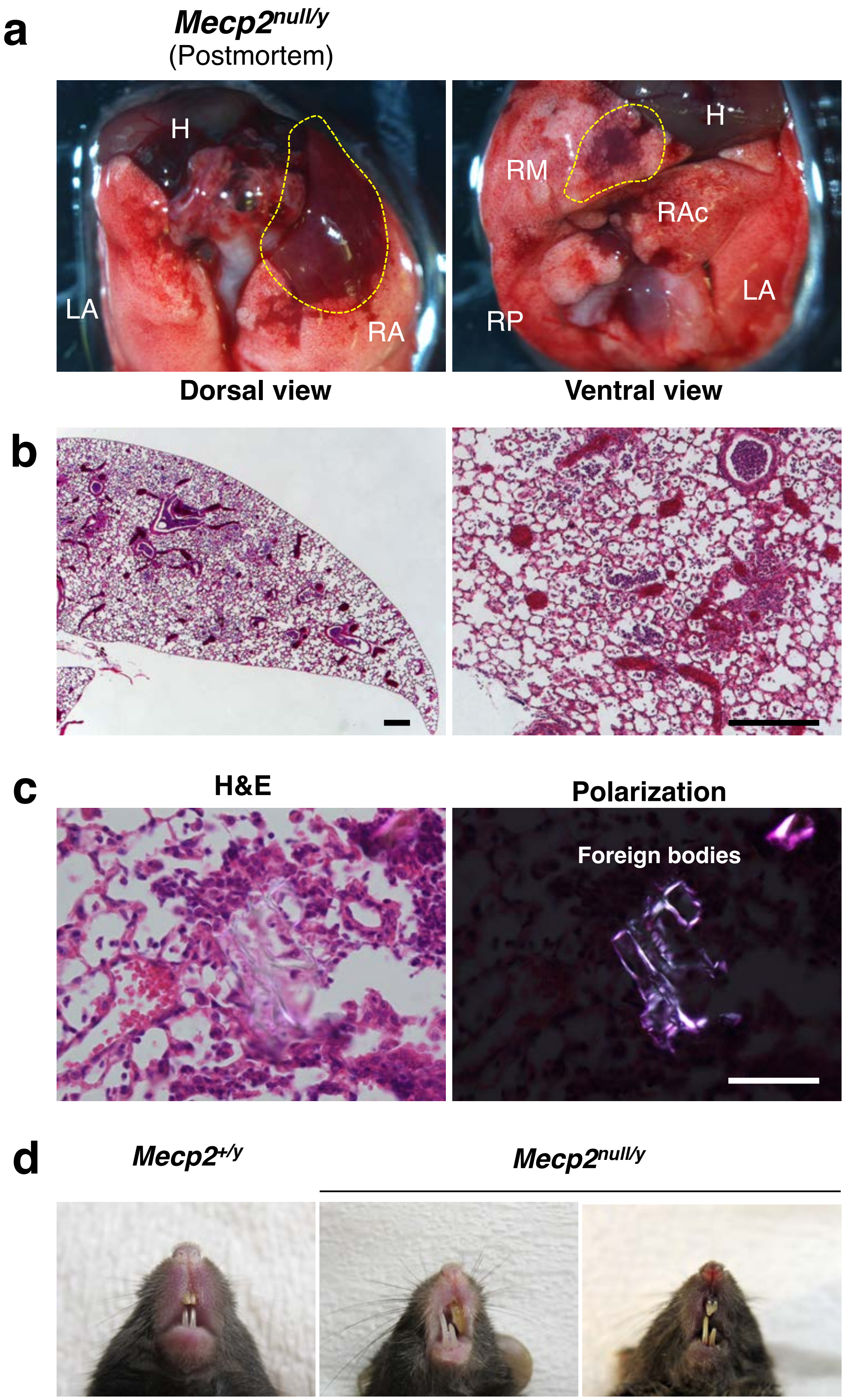
a
Mecp2null/y
(Postmortem)
H
LA
RA
H
RM
RAc
LA
RP
Dorsal view
Ventral view
b
c
H&E
Polarization
Foreign bodies
d
Mecp2+/y
Mecp2null/y

Figure S2

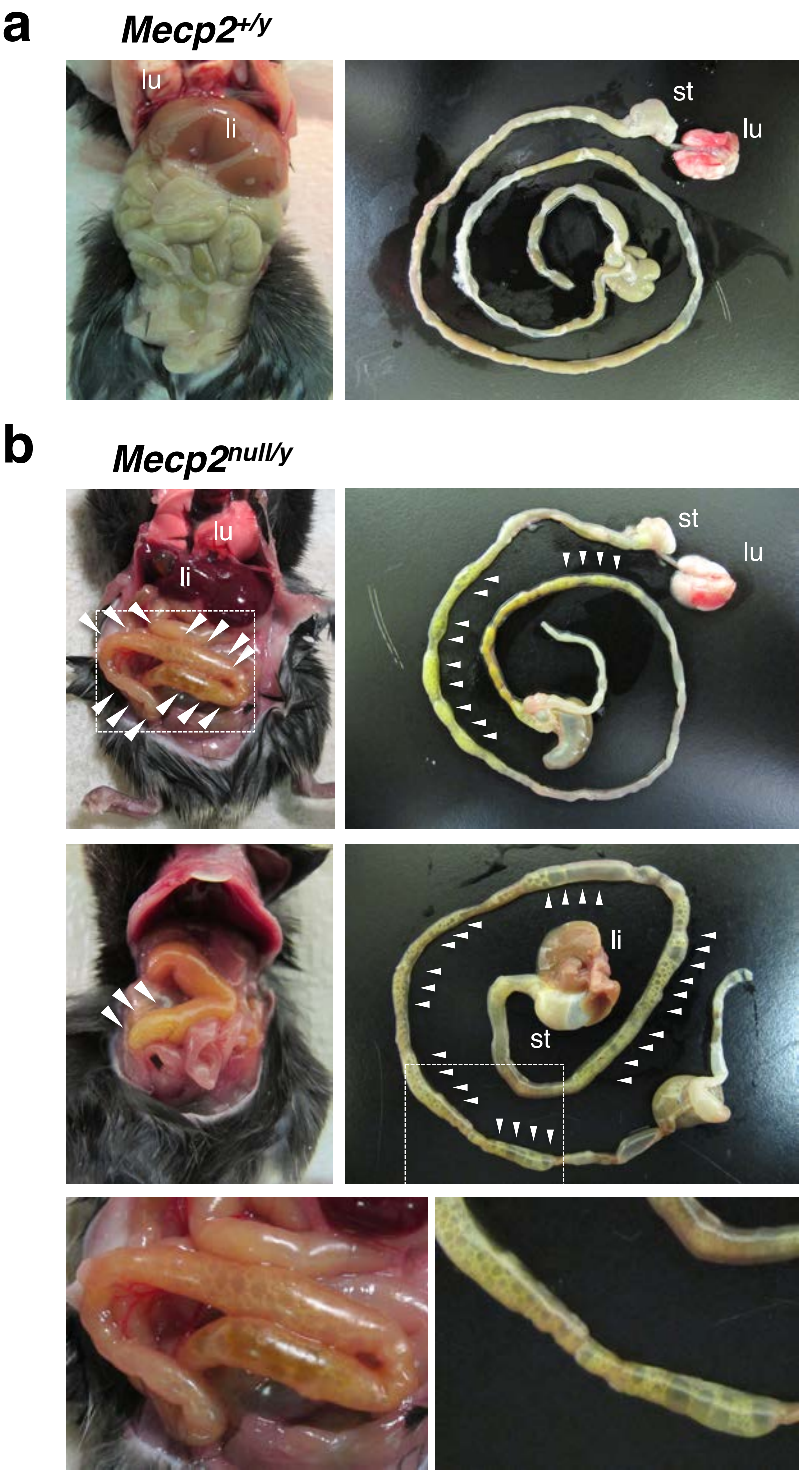

Figure S3

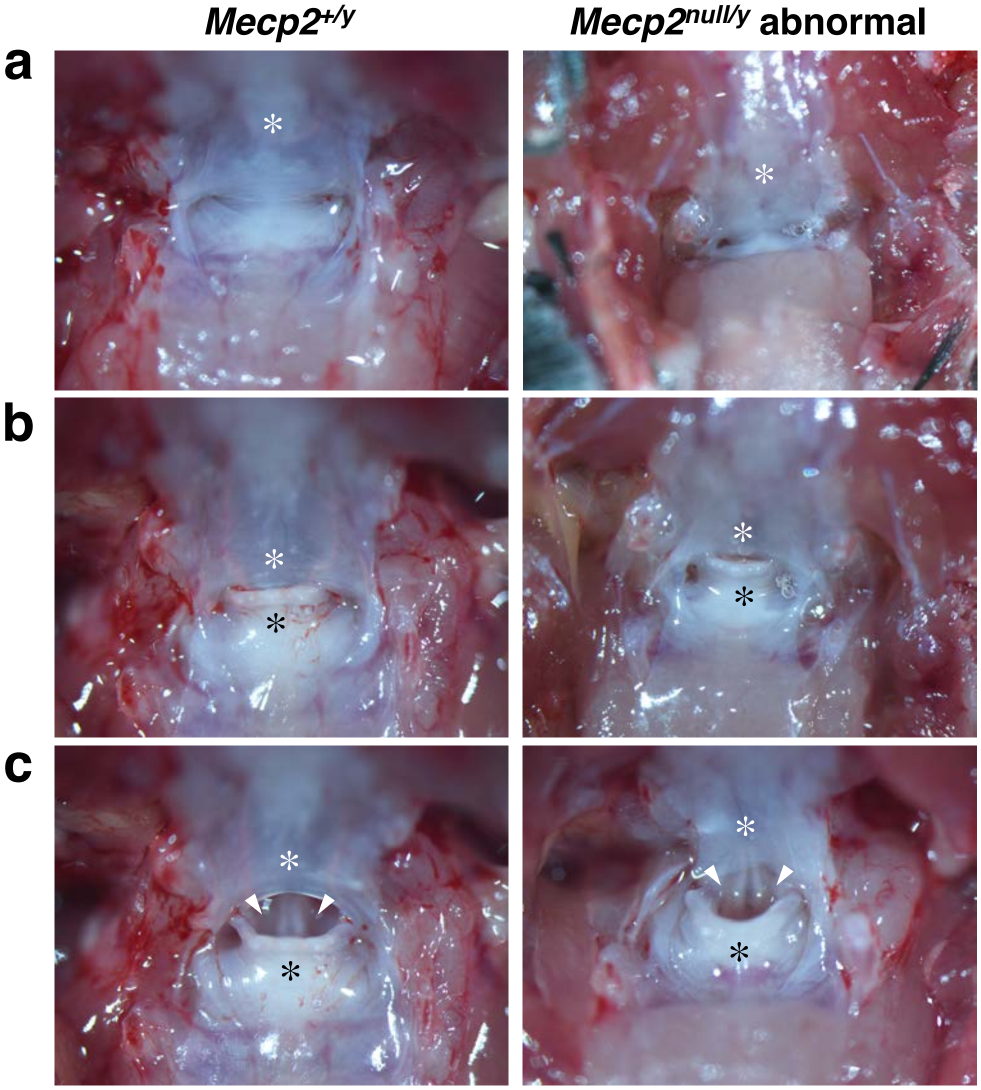

Figure S4

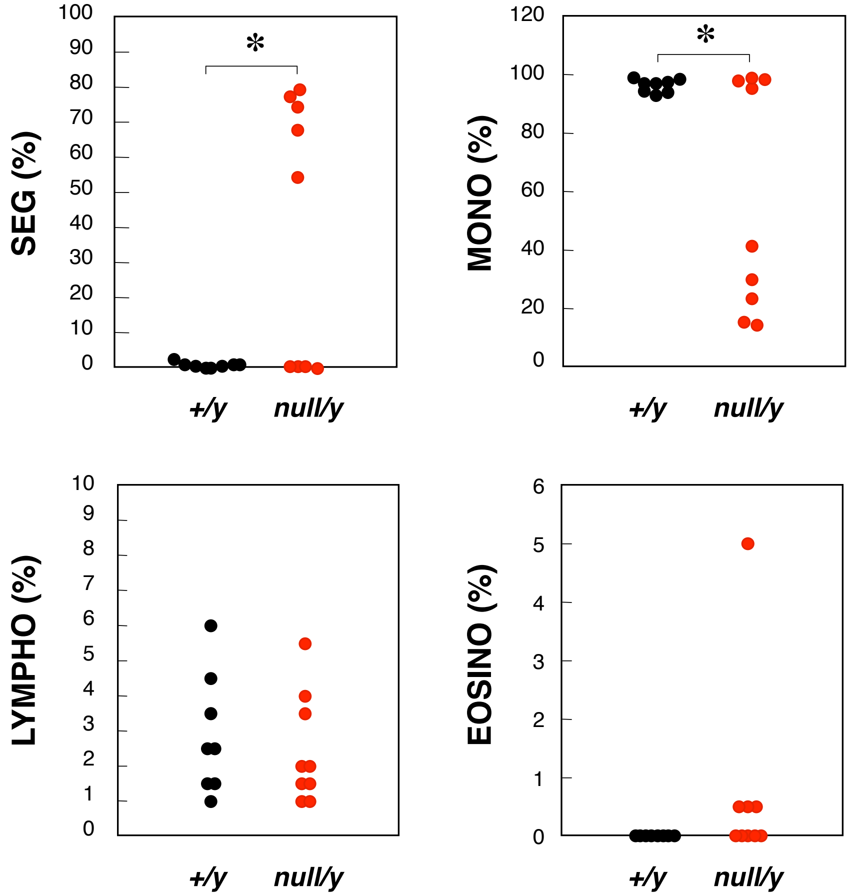

SEG (%)
100
90
80
70
60
50
40
30
20
10
0
*
+/y
null/y
MONO (%)
120
100
80
60
40
20
0
*
+/y
null/y
LYMPHO (%)
10
9
8
7
6
5
4
3
2
1
0
+/y
null/y
EOSINO (%)
6
5
4
3
2
1
0
+/y
null/y

Figure S5

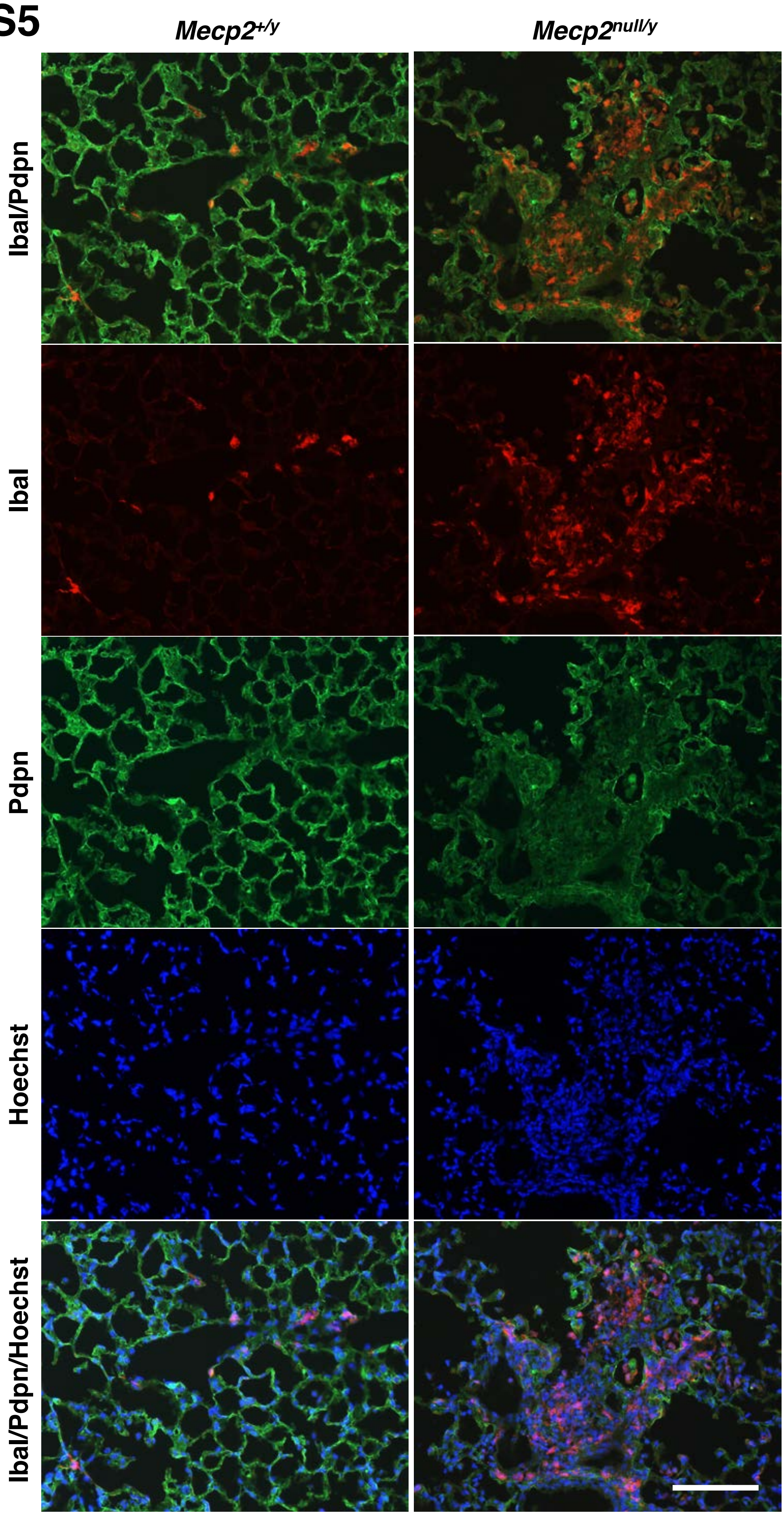

Mecp2+/y
*Mecp2*null/y
IbaI/Pdpn
IbaI
Pdpn
Hoechst
IbaI/Pdpn/Hoechst

Figure S6

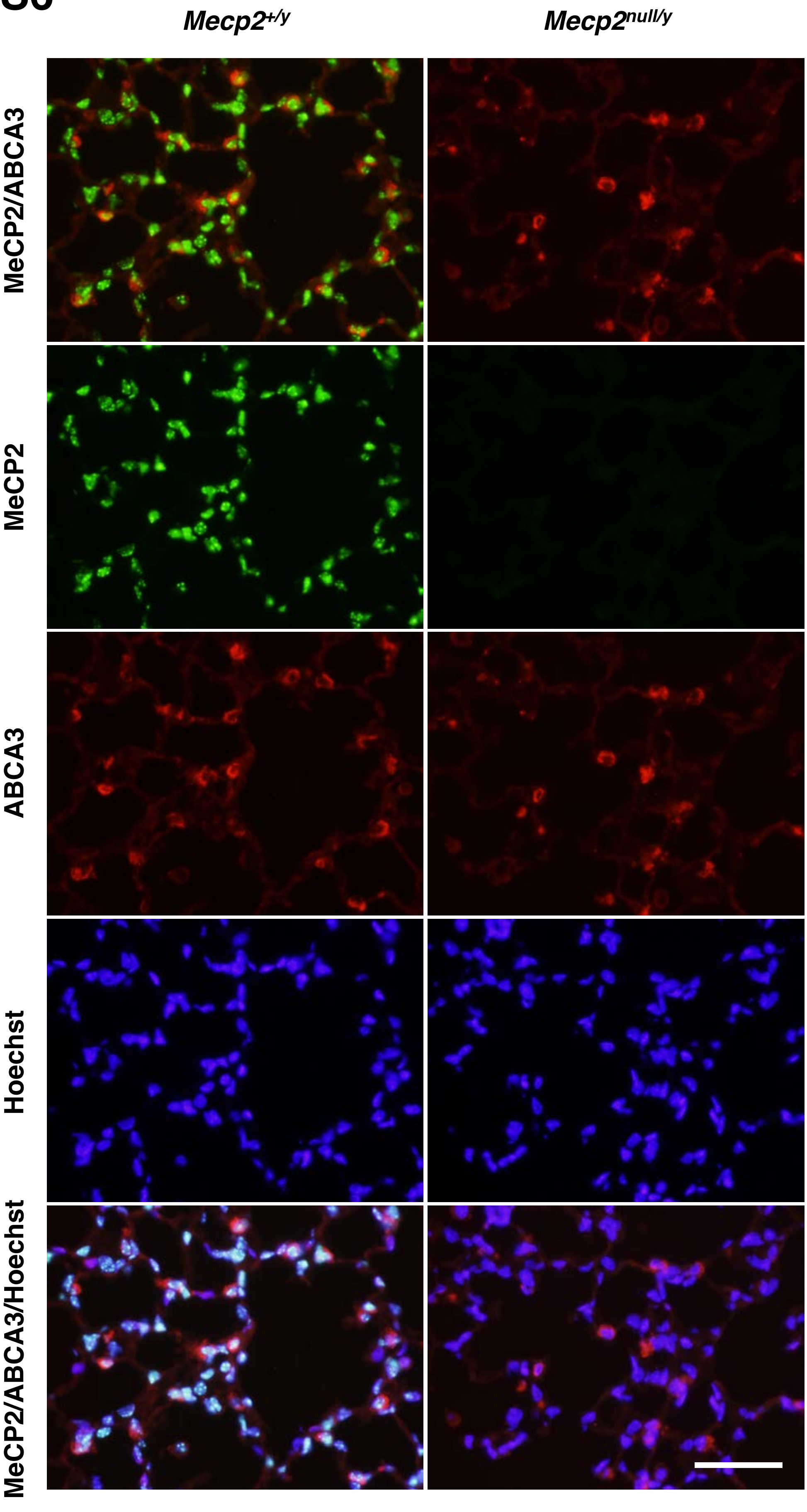

Mecp2+/y
*Mecp2*null/y
MeCP2/ABCA3
MeCP2
ABCA3
Hoechst
MeCP2/ABCA3/Hoechst

Figure S7

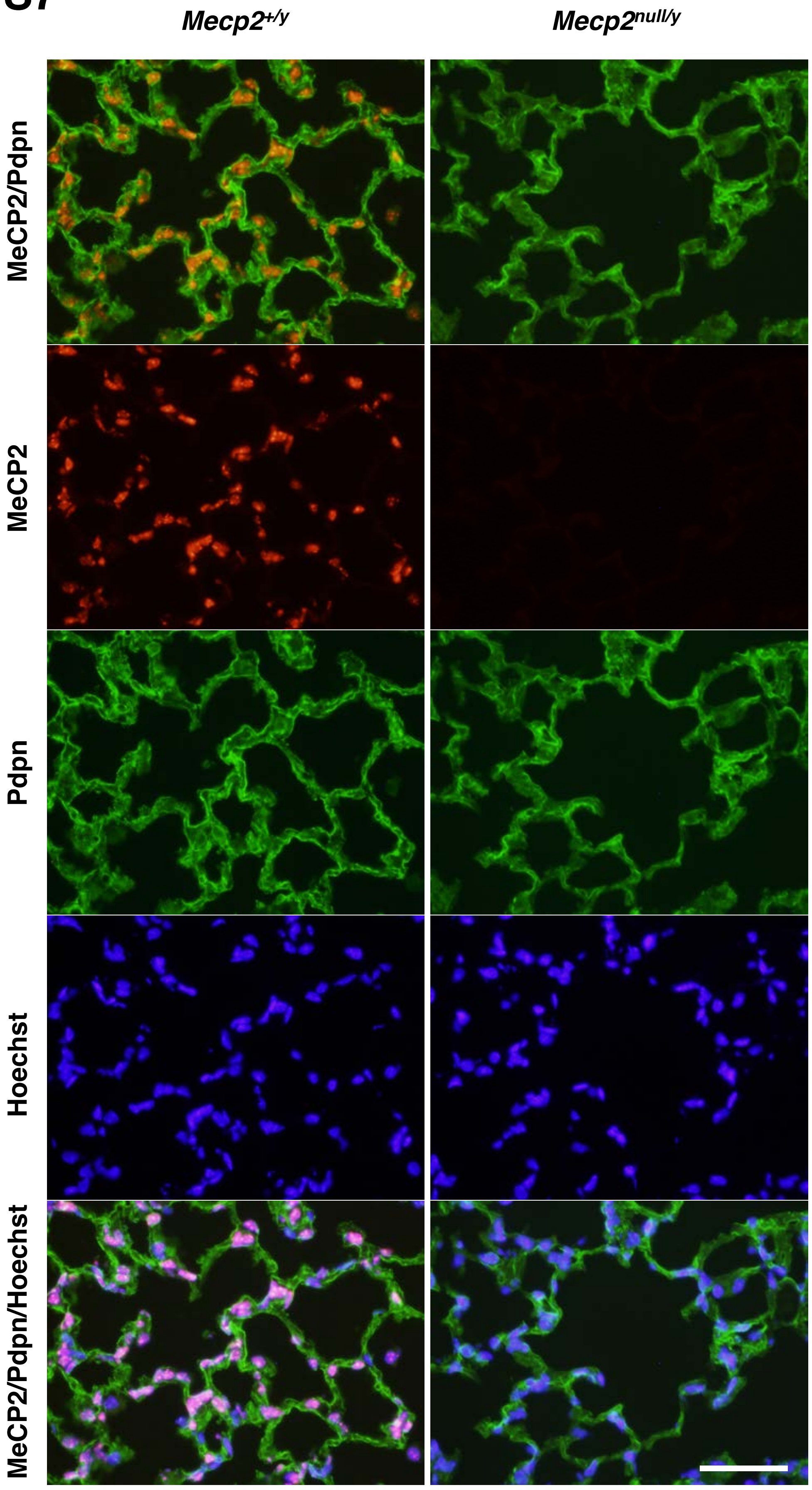

Mecp2+/y
*Mecp2*null/y
MeCP2/Pdpn
MeCP2
Pdpn
Hoechst
MeCP2/Pdpn/Hoechst

Figure S8

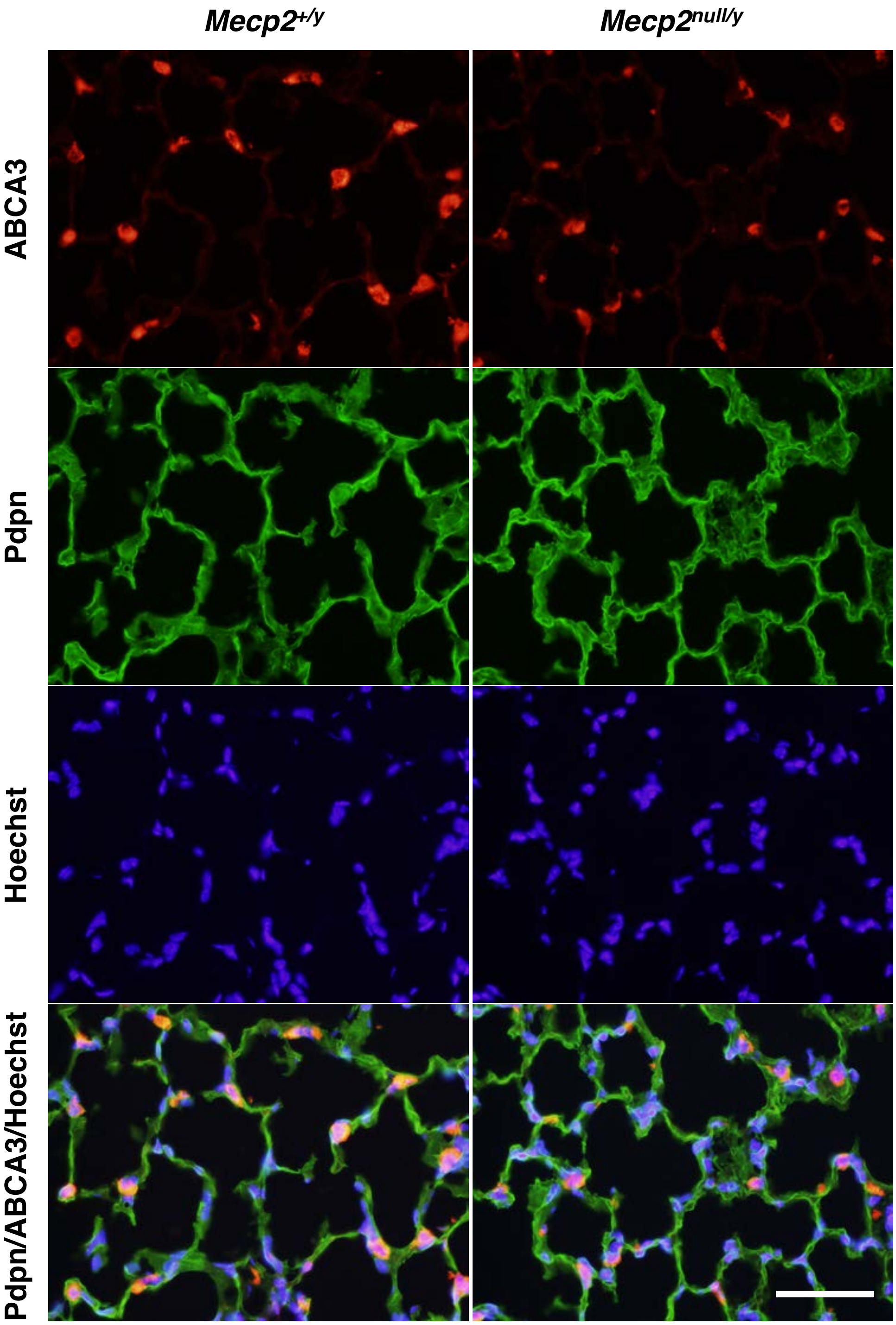

Figure S9

a

anesthetized

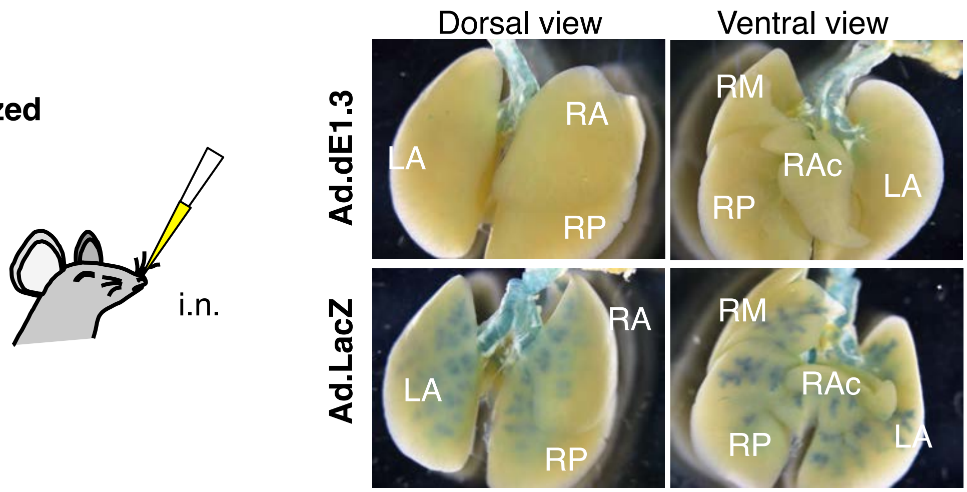

b

awake

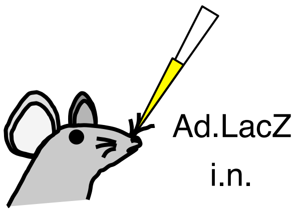

**awake**	**Ad.LacZ**
Mecp2$^{+/y}$	1/14 (7.1 %)
Mecp2$^{null/y}$	9/14 (64.3 %)

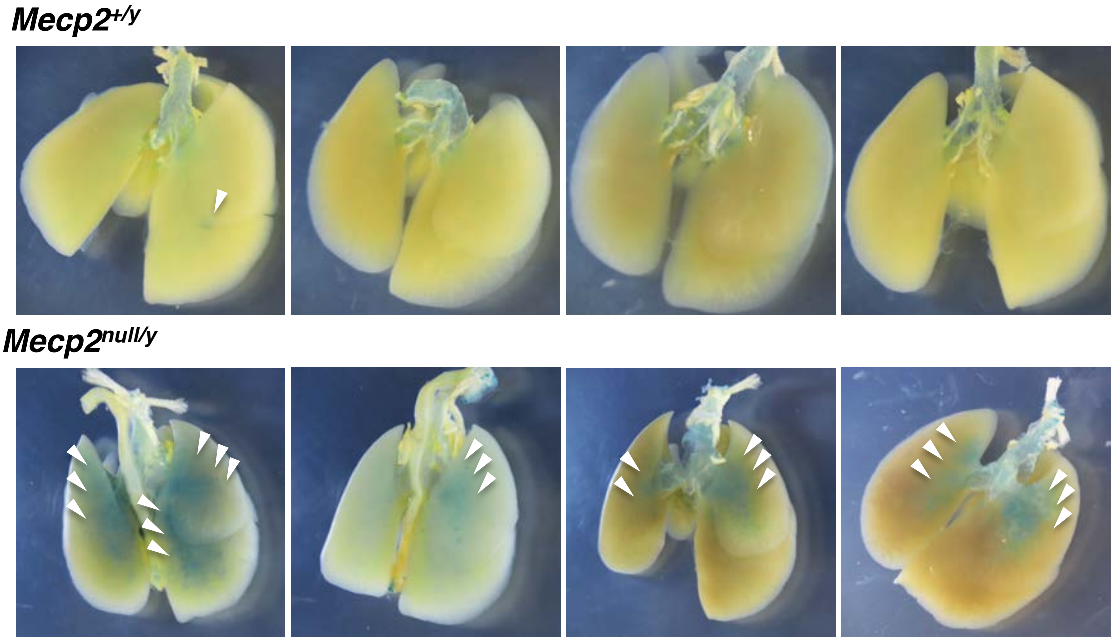

Figure S10

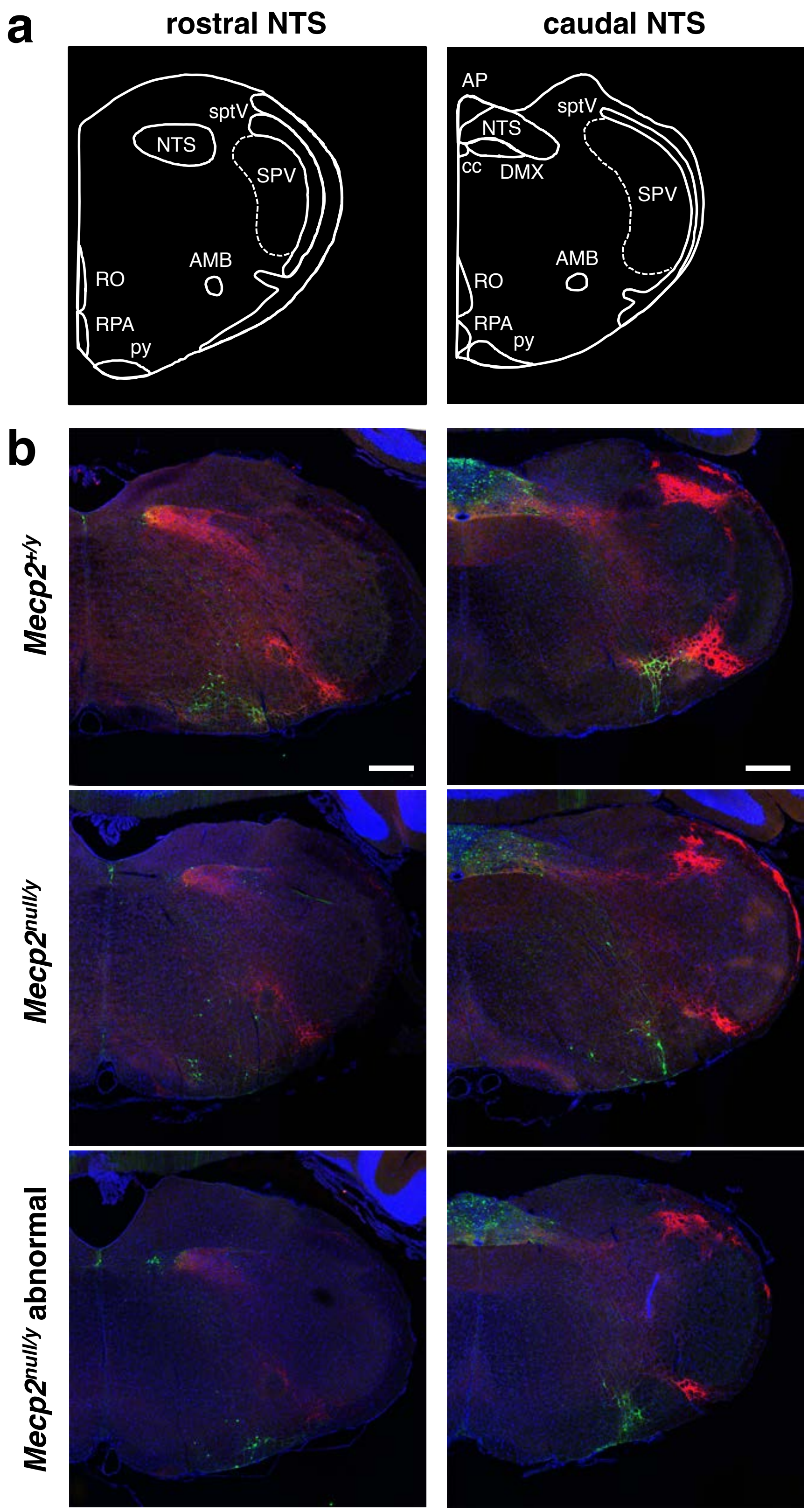
a
rostral NTS
caudal NTS
sptV
NTS
SPV
AMB
RO
RPA
py
AP
sptV
NTS
cc
DMX
SPV
AMB
RO
RPA
py
b
Mecp2+/y
Mecp2null/y
Mecp2null/y abnormal

Figure 11s

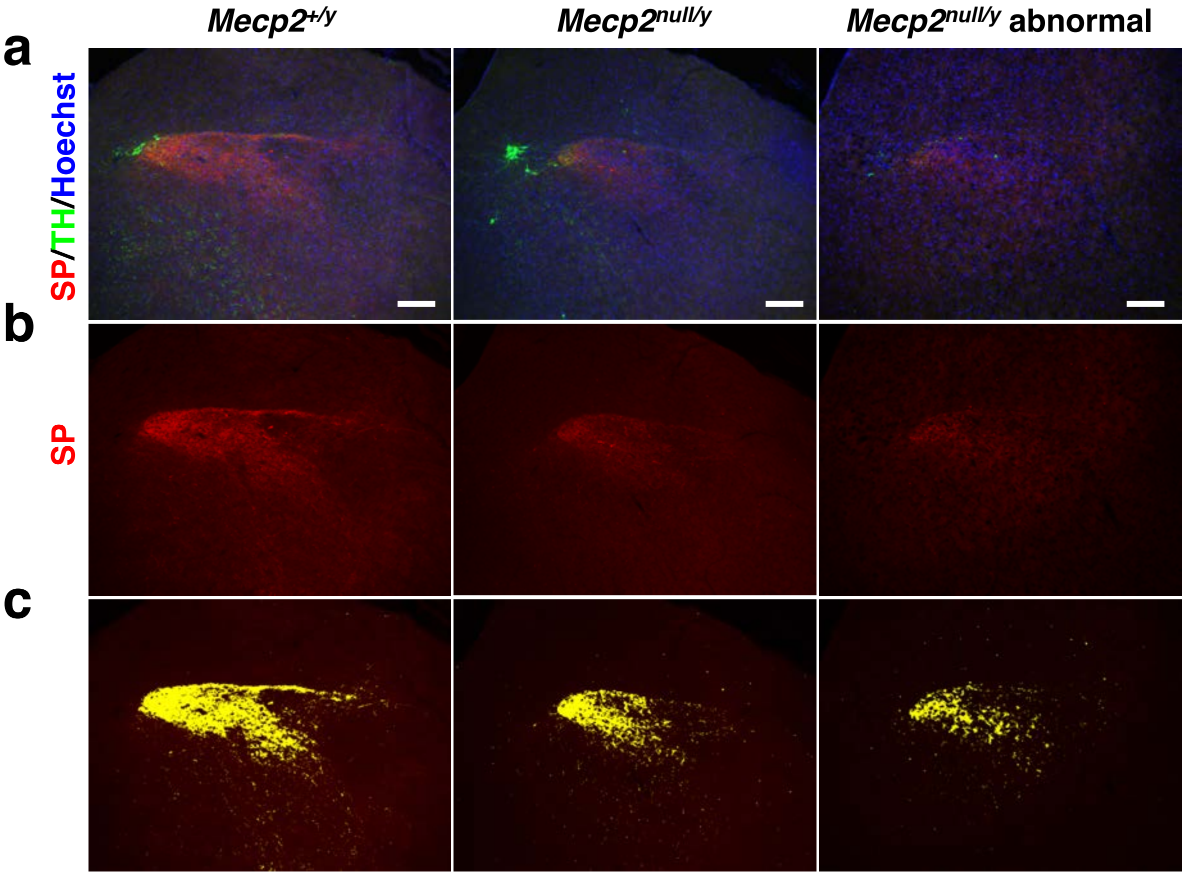

Mecp2+/y
*Mecp2*null/y
*Mecp2*null/y abnormal
a
b
c
SP/TH/Hoechst
SP

Figure 12s

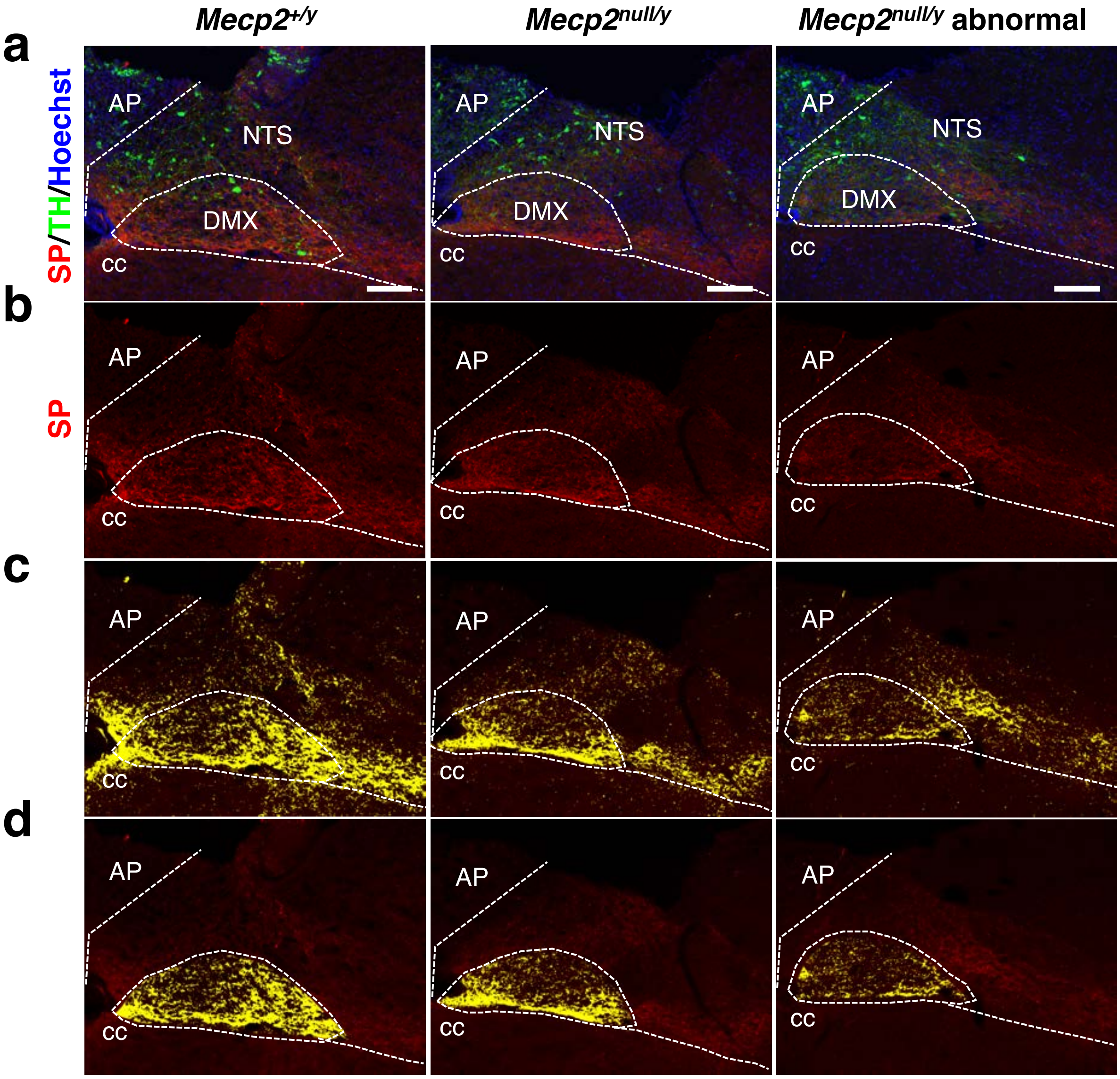

Mecp2+/y
Mecp2null/y
Mecp2null/y abnormal
a
b
c
d
SP/TH/Hoechst
SP
AP
NTS
DMX
cc
